# White matter alterations in Attention-Deficit/Hyperactivity Disorder (ADHD): a systematic review of 129 diffusion imaging studies with meta-analysis

**DOI:** 10.1038/s41380-023-02173-1

**Published:** 2023-07-21

**Authors:** Valeria Parlatini, Takashi Itahashi, Yeji Lee, Siwei Liu, Thuan T. Nguyen, Yuta Y. Aoki, Stephanie J. Forkel, Marco Catani, Katya Rubia, Juan H. Zhou, Declan G. Murphy, Samuele Cortese

**Affiliations:** 1 Sackler Institute of Translational Neurodevelopment, Department of Forensic and Neurodevelopmental Sciences, Institute of Psychiatry, Psychology and Neuroscience, King’s College London, SE5 8AF London, UK; 2 Department of Forensic and Neurodevelopmental Sciences, Institute of Psychiatry, Psychology and Neuroscience, King’s College London, SE5 8AF London, UK; 3 Department of Child and Adolescent Psychiatry, Institute of Psychiatry, Psychology and Neuroscience, King’s College London, SE5 8AF London, UK; 4 Medical Institute of Developmental Disabilities Research, Showa University, 6-11-11 Kita-karasuyama, Setagaya-ku, Tokyo, Japan; 5 Center for Neuroscience Imaging Research, Institute for Basic Science (IBS), Sungkyunkwan University (SKKU), Suwon, 16419 Republic of Korea; 6 Centre for Sleep and Cognition, Yong Loo Lin School of Medicine, National University of Singapore, Singapore, Singapore; 7 Centre for Translational Magnetic Resonance Research, Yong Loo Lin School of Medicine, National University of Singapore, Singapore, Singapore; 8 Integrative Sciences and Engineering Programme, National University of Singapore, Singapore, Singapore; 9 Department of Psychiatry, Aoki Clinic, Tokyo, Japan; 10 Donders Centre for Brain, Cognition and Behaviour, Radboud University, Nijmegen, The Netherlands; 11 Centre for Neuroimaging Sciences, Department of Neuroimaging, Institute of Psychiatry, Psychology and Neuroscience, King’s College London, London, UK; 12 Brain Connectivity and Behaviour Laboratory, Sorbonne Universities, Paris, France; 13 Departments of Neurosurgery, Technical University of Munich School of Medicine, Munich, Germany; 14 Department of Electrical and Computer Engineering, National University of Singapore, Singapore, Singapore; 15 Centre for Innovation in Mental Health, School of Psychology, Faculty of Environmental and Life Sciences, University of Southampton, Southampton, UK; 16 Clinical and Experimental Sciences (CNS and Psychiatry), Faculty of Medicine, University of Southampton, Southampton, UK; 17 Solent NHS Trust, Southampton, UK; 18 Hassenfeld Children’s Hospital at NYU Langone, New York University Child Study Center, New York, NY USA; 19 Division of Psychiatry and Applied Psychology, School of Medicine, University of Nottingham, Nottingham, UK

**Keywords:** Neuroscience, ADHD

## Abstract

Aberrant anatomical brain connections in attention-deficit/hyperactivity disorder (ADHD) are reported inconsistently across diffusion weighted imaging (DWI) studies. Based on a pre-registered protocol (Prospero: CRD42021259192), we searched PubMed, Ovid, and Web of Knowledge until 26/03/2022 to conduct a systematic review of DWI studies. We performed a quality assessment based on imaging acquisition, preprocessing, and analysis. Using signed differential mapping, we meta-analyzed a subset of the retrieved studies amenable to quantitative evidence synthesis, i.e., tract-based spatial statistics (TBSS) studies, in individuals of any age and, separately, in children, adults, and high-quality datasets. Finally, we conducted meta-regressions to test the effect of age, sex, and medication-naïvety. We included 129 studies (6739 ADHD participants and 6476 controls), of which 25 TBSS studies provided peak coordinates for case-control differences in fractional anisotropy (FA)(32 datasets) and 18 in mean diffusivity (MD)(23 datasets). The systematic review highlighted white matter alterations (especially reduced FA) in projection, commissural and association pathways of individuals with ADHD, which were associated with symptom severity and cognitive deficits. The meta-analysis showed a consistent reduced FA in the splenium and body of the corpus callosum, extending to the cingulum. Lower FA was related to older age, and case-control differences did not survive in the pediatric meta-analysis. About 68% of studies were of low quality, mainly due to acquisitions with non-isotropic voxels or lack of motion correction; and the sensitivity analysis in high-quality datasets yielded no significant results. Findings suggest prominent alterations in posterior interhemispheric connections subserving cognitive and motor functions affected in ADHD, although these might be influenced by non-optimal acquisition parameters/preprocessing. Absence of findings in children may be related to the late development of callosal fibers, which may enhance case-control differences in adulthood. Clinicodemographic and methodological differences were major barriers to consistency and comparability among studies, and should be addressed in future investigations.

## Introduction

Attention-deficit/hyperactivity disorder (ADHD) is a neurodevelopmental condition characterized by age-inappropriate inattentive and/or hyperactive-impulsive symptoms [1]. Cognitively, individuals with ADHD may present with deficits in executive functions, such as motor inhibition, attention, and/or working memory [2]. It is commonly diagnosed in childhood, with community prevalence between 2-7% [3], but its impairing symptoms persists in adulthood in up to 65% of cases, and are associated with poor social and occupational outcomes [4]. Co-occurrent disorders, from autism spectrum disorder (ASD) to affective and substance abuse disorders, are often observed [5, 6]. Multiple genetic and environmental factors contribute to ADHD, but it is unclear how they interplay with brain development to produce symptoms and cognitive deficits [2]. Understanding the underlying neuropathophysiology is crucial to develop and tailor behavioral, pharmacological or brain-based treatments.

Meta-analyses of structural and functional neuroimaging studies have identified several case-control differences, but mainly focused on regional alterations [2]. However, brain regions operate as neural networks, and there is increasing evidence that anatomical brain connections are also affected in ADHD [7]. Diffusion-weighted imaging (DWI) is the only non-invasive imaging method that allows us to study the anatomy of brain connections in the living human. It measures the diffusion of water molecules, which in the brain is restricted by structures such as myelin and axons, providing information on the microstructural organization of white matter tracts [8, 9] (Box 1).

The first systematic review of diffusion imaging studies in ADHD was published in 2012 and included 15 studies, mostly in pediatric samples [10]. The meta-analysis of nine of the included studies revealed diffuse alterations mainly affecting fronto-striato-cerebellar connections [10]. The two following meta-analyses, respectively published in 2016 and 2018, included VBA and/or TBSS studies and mainly identified regions of reduced FA in posterior commissural fibers [11, 12]. The separate analysis of TBSS and VBA studies also allowed the identification of regions of increased FA in the corpus callosum (CC) and cingulum [11]. These evidence syntheses were important to elucidate the most consistent findings in ADHD. Still, they did not consider studies using techniques not amenable to meta-analysis, and included a very limited number of studies in adults. Since then, DWI has benefitted from considerable technological advances, and the quality and number of published studies in the field of ADHD has progressively increased. A more comprehensive review with meta-analysis is therefore timely to provide a broader view of the findings, identify the most robust evidence, and highlight methodological considerations. Therefore, we conducted a comprehensive systematic review of DWI studies in ADHD, including a quality assessment of imaging data acquisition, preprocessing and analysis. We then performed, wherever possible, meta-analyses including individuals with ADHD of any age and, separately, children and adults. Finally, we conducted meta-regressions to test the effect of age, sex, and medication-naïve status, followed by a sensitivity analysis including only high-quality datasets.

BOX 1 Traditional (tensor based) DWI studies in ADHD have mainly analyzed case-control differences using four different approaches: voxel-based analysis (VBA), region of interest (ROI) analysis, tract-based spatial statistics (TBSS), and tractography. The first diffusion imaging study in ADHD [19] used a VBA approach, which offers the advantage of identifying whole-brain white matter alterations without a priori anatomical hypotheses. Nevertheless, this approach is affected by the choice of normalization and interpolation techniques, and the need for multiple comparisons correction may reduce the power to detect significant group differences [146–149]. Conversely, ROI approaches extract diffusion measurements from circumscribed brain areas selected based on a priori hypotheses. Thus, they are sensitive to potential differences in the selected regions but may be limited by the manual selection of ROIs, especially in early studies. Further, they are not amenable to be included in whole-brain meta-analytic syntheses, as they could inflate group differences in the selected regions [150, 151]. TBSS has been developed to partly overcome the limitations of both VBA and ROI methods [152]. It uses a whole-brain white matter ‘skeleton’ mask to restrict the analyses to the central part of the major tracts. This minimizes potential misalignment artefacts while increasing the statistical power to detect group differences. However, TBSS may not capture potential differences in the periphery of a tract and is still based on comparisons at the voxel level. Finally, tractography allows the analysis of group differences along specific white matter tracts [149]. Its main advantage is the possibility of extracting diffusion measurements from the entire course of individual tracts, although it may be less accurate in regions with complex fiber organization (e.g., crossing fibers) [153]. Further, the high variability among tractography algorithms and the placement of the ROIs used to identify the tracts may limit comparisons among studies. DWI allows the extraction of quantitative indexes of white matter microstructural organization, such as fractional anisotropy (FA), mean diffusivity (MD), and axial and radial diffusivity (AD and RD, respectively) [154, 155]. FA ranges from 0 to 1, according to the degree of tissue anisotropy, and is the most used DWI metric. MD represents the average diffusion, whilst AD and RD respectively reflect diffusion along the main axis and perpendicular to it [156]. Although there is not a precise correspondence between these measures and the underlying biological attributes, multiple aspects of tissue microstructure, including the number and size of axons, myelination, and membrane permeability may contribute to FA. Similarly, axon size and density may affect AD, whilst myelination may contribute to RD [127, 156]. As these indices vary during brain maturation and in pathological conditions [157, 158], they are useful for case-control comparisons or to study age-related changes.

## Methods

This study followed a preregistered protocol (PROSPERO 2021 CRD42021259192) and is reported in line with the 2020 Preferred Reporting Items for Systematic Reviews and Meta-Analyses (PRISMA) Statement [13].

### Data sources

We searched the following electronic databases: PubMed (Medline), Ovid databases (Ovid MEDLINE^®^, EMBASE Classic+EMBASE, PsycINFO), and Web of Knowledge (including Web of Science, Biological Abstracts, BIOSIS, Food science and technology abstracts), without language and date restrictions. The search was first conducted on the 22^nd^ June 2021 and updated on the 26^th^ March 2022. Search terms and syntax for each electronic database are reported in the Supplementary material. The reference lists of previous reviews were hand-searched for any additional eligible studies that could have been missed in the electronic searches.

### Identification and selection of studies

First, two authors (VP and TI) independently screened titles and abstracts of all nonduplicated papers and agreed on a final list of studies that proceeded to full-text screening. Then, these two authors independently assessed the eligibility of these studies for the systematic review and meta-analysis. Any discrepancy between the two authors was resolved by a third senior author (SC).

### Study selection

Studies were included in the systematic review if they:

1. were peer-reviewed, indicating methodological adequacy, in line with recent meta-analyses [11, 12];
2. recruited individuals diagnosed with ADHD based on the criteria of the Diagnostic and Statistical Manual of Mental Disorders (DSM-III or following editions) or International Classification of Diseases (ICD-9 or 10) and typically developing (TD) controls;
3. collected diffusion imaging data from both ADHD participants and TD controls.

According to our pre-registered protocol, we assessed the feasibility of conducting a meta-analysis of eligible whole-brain studies (i.e., TBSS and VBA studies), provided their number was increased sufficiently (i.e., by 50%), as compared to the last published meta-analysis before the start of this study [11], to justify a new meta-analysis. Additionally, studies were eligible for the meta-analysis only if they compared whole-brain diffusion imaging data (any metric) between individuals with ADHD and TD controls.

### Data extraction

Two authors (TI and YL) independently extracted information from the studies selected for the systematic review. Any discrepancy between the two authors was resolved by a third author (VP). Data extracted from all studies in the systemic review included: sample size, demographic and clinical characteristics (i.e., age, sex, total intelligent quotient (IQ), ADHD presentation, comorbidities, and medication-naïve status); analytic method; significant case-control comparisons and associations between diffusion metrics and symptom severity or cognitive performance. Further, data on imaging data acquisition, preprocessing and analysis were extracted for the quality assessment (see below). Authors were contacted for missing data on imaging parameters included in the quality assessment.

For the meta-analyses, we extracted peak coordinates and their effect sizes for FA, MD, AD and RD contrasts. In studies not providing exact effect sizes for peak coordinates, the study threshold for significance was interpreted as the effect size, as in previous reports [11]. In cases where peak coordinates and/or effect sizes were not provided, we contacted the corresponding authors to obtain the missing data. *P*-values of peak coordinates were converted to t-values using the anisotropic effect size signed differential mapping (AES-SDM) utility (http://www.sdmproject.com/utilities/?show=Statistics) [14]. Peak coordinates are available as Supplementary material.

### Quality assessment

In the absence of an established tool to rate the quality of DWI studies, criteria were identified based on published recommendations (listed in Supplementary Table S3). A traffic light system was then implemented, and studies were assigned a low/medium/high-quality rating separately for imaging data acquisition, preprocessing, and analysis, as well as an overall rating based on the worse single rating. Two authors (SL and TTN) independently completed the quality assessment and discrepancies were resolved by a third author (JHZ).

### Meta-analysis and meta-regressions

We ran meta-analyses for diffusion metrics that had peak coordinates available from at least five suitable studies. As in previous meta-analyses [11, 12], we used the Signed Differential Mapping (SDM) software, version 6.21 [15] (https://www.sdmproject.com/), to analyze regional differences in tract metrics between ADHD and TD control groups; and used the TBSS template for TBSS studies. Also consistently with prior studies [11, 12], we used a random effects model, and the same statistical threshold that was previously applied (*p* < 0.005 at the voxel level with an extent threshold of 10 voxels). Meta-analyses were repeated in the pediatric sample (i.e., children/adolescents <18 years) and adults separately; and then in children (<12 years) and adolescents separately (Supplementary Table S2). We also conducted a post-hoc sensitivity analysis only including datasets judged of high quality. Finally, we ran meta-regression analyses to test the linear effects of age, percentage of males and medication-naïve subjects. We chose these variables because they have been associated with variation in white matter characteristics [16, 17]. Further, given that a previous report suggested that stimulant treatment could affect FA measures in children, but not adults [16], we tested the potential confounding effect of treatment exposure in the meta-regression of age.

## Results

### Systematic review

**Fig. 1:**
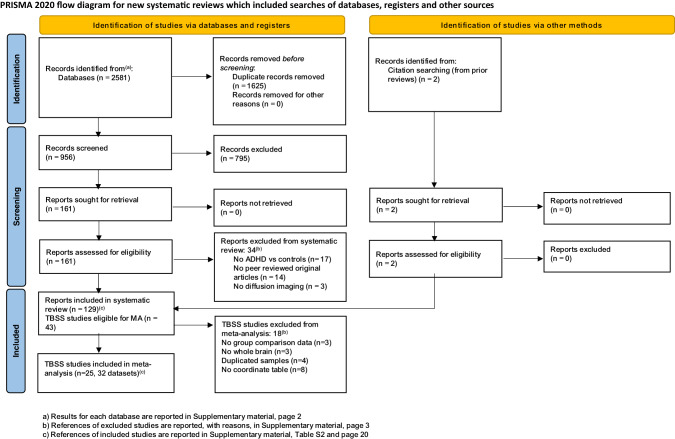
PRISMA 2020 flow diagram.

**Fig. 2:**
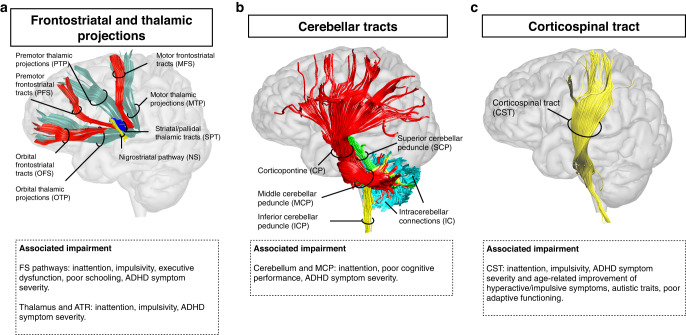
Projection pathways and associated impairment. This figure displays the distinct components of the frontostriatal and thalamo-frontal connections (panel **a**); cerebellar pathways (panel **b**); and the corticospinal tract (panel **c**). Significant associations between tract metrics and cognitive deficits or symptoms are reported. Additional abbreviations: ATR anterior thalamic radiation, FS frontostriatal tract.

**Fig. 3:**
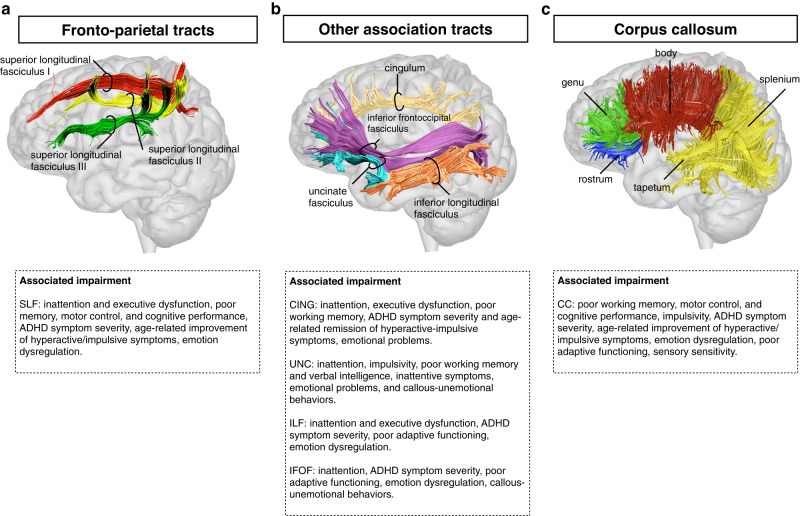
Association and commissural pathways and associated impairment. This figure shows the three branches of the superior longitudinal fasciculus (SLF) (Panel **a**); the cingulum (CING), uncinate (UNC), inferior longitudinal fasciculus (ILF) and inferior fronto-occipital fasciculus (IFOF) (Panel **b**); and the main subdivisions of the corpus callosum (CC) (Panel **c**). Significant associations between tract metrics and cognitive deficits or symptoms are reported.

As shown in the PRISMA flow diagram (Fig. 1), from a pool of 956 possibly relevant references, we included 129 studies (96 in children, 25 in adults and 8 including both age groups), for a total of 6739 ADHD participants and 6476 controls. As many studies in pediatric samples included both children and adolescents, in the narrative synthesis we aggregated them under ‘children’ (i.e. <18 years). However, in the meta-analysis, we also considered them separately (Supplementary Table S2). With regards to the imaging data analytic approach, the retained studies used one or a combination of the following: TBSS (43 studies), tractography (38 studies), ROI (22 studies), VBA (16 studies), network/graph analysis (17 studies) or other techniques (e.g., fixel-based analysis) (3 studies). Here, we first summarize the results of these studies as a narrative review, according to the topographical organization of the main brain connections [18]. These can be grouped in projection (cortico-subcortical) pathways, which relay sensory-motor information; association (intra-hemispheric) pathways, which integrate functions of brain regions within the same hemisphere; and commissural (inter-hemispheric) pathways, which support information transfer between the two hemispheres [18]. Associations between diffusion metrics and symptoms/cognitive deficits are summarized in Figs. 2 and 3 and detailed in Supplementary material (page 5). Detailed characteristics of included studies are reported in Tables 1 and 2. Excluded studies are reported, with reasons, in the Supplementary material (page 3 and Supplementary Table S1). We then discuss the quality assessment and present the results of the meta-analyses and meta-regressions.

**Table 1:** Diffusion imaging studies in children with ADHD.

Study	ADHD participants							Control participants				Imaging method	Main findings
	*N*	Age	Males (*N*)	IQ	ADHD presentation (*N*)	Comorbidities (*N*)	Drug naïve (*N*)	*N*	Age	Males (*N*)	IQ		ADHD vs controls and associations
Acer et al., 2017	17	10.55 (2.8)	15	101.3 (12.3)	NA	0	NA	10	10.21 (2.1)	7	108.6 (8.2)	ROI	Increased MD in the deep GM, amygdala, thalamus, substantia nigra, and cerebellum bilaterally. Increased RD in the deep GM, caudate, thalamus, substantia nigra and hippocampus bilaterally. Increased FA in the bilat splenium of the CC
Adisetiyo et al., 2014	22	12.6 (2.8)	15	106.8 (15.6)	ADHD-C: 14 ADHD-I: 8	11	12	27	13.3 (2.6)	12	111.3 (14.7)	TBSS + VBA	Greater WM microstructural complexity in bilat frontal and parietal lobes (including SLF, ACR, superior and posterior CR, PTR, left CING), temporal lobes (ILF, IFOF, PTR), L occipital lobe (SLF, PTR), thalamus, insula, CC, and R IC and EC
Alger et al., 2021	1)ADHD+PAE: 23 2) ADHD-PAE: 19	1) 9.7 (1.5) 2) 10.7 (0.9)	1) 16 2) 12	1) 94.9 (12.5) 2) 107.5 (11.2)	NA	0	1) 10 2) 14	28	11.1 (1.5)	13	116.7 (15.4)	ROI	Lower global MD in ADHD without prenatal alcohol exposure
Ameis et al., 2016	31	10.3 (1.8)	25	103.4 (12.6)	NA	23	18	62	10.8 (2.8)	37	112.5 (17.1)	TBSS	Reduced FA in the genu and splenium of the CC, CST, ILF, arcuate, and IFOF. ASSOC: FA and adaptive functioning
Aoki et al., 2017	55	9.5 (1.5)	41	110 (14)	ADHD-C: 36 ADHD-I: 16 NOS: 3	25	41	50	9.4 (1.5)	38	114 (13)	TBSS	No group differences. ASSOC: AD in the CC and inattentive symptoms
Ashtari et al., 2005	18	8.94 (1.5)	12	101.89 (20.9)	ADHD-C:18	8	6	15	9.13 (1.4)	9	105.07 (10.0)	VBA	Reduced FA in R premotor, anterior limb IC and cerebral peduncle; L MCP, cerebellum, and parieto-occipital areas
Ball et al., 2019	70	[9.4-11.9]	NA	NA	NA	NA	47	90	[9.4-11.9]	NA	NA	TBSS + multimodal	ASSOC: FA in the fornix, SLF and genu of the CC with hyperactivity
Basay et al., 2016	71	10.88 (1.36)	59	109.42 (18.60)	NA	11	1	24	10.80 (2.02)	18	111.45 (17.84)	TBSS	Reduced FA in the L CING and R retrolenticular IC
Beare et al., 2017	21	13.28 (1.86)	21	96.95 (10.15)	ADHD-C: 21	NA	15	21	14.79 (2.12)	21	111.76 (9.66)	TG + network	Increased connectivity in subnetwork including bilat frontal regions, CING, and putamen, and extending to L fronto-temporo-parieto-occipital regions, thalamus and pallidum. ASSOC: FA cluster strength and symptom severity
Bechtel et al., 2009	1) epilepsy+ ADHD: 8 2) ADHD: 14	1) 11.63 (1.69) 2) 10.43 (1.34)	1) 8 2) 1 4	NA	ADHD-I: 1) 4 2) 5	8	0	12	10.92 (1.64)	12	NA	ROI	Reduced FA in the R MCP
Bessette et al., 2019	67	15.41 (1.78)	55	105.28 (12.06)	ADHD-C:67	29	25	68	15.43 (1.73)	50	108.05 (9.25)	TBSS	ASSOC: FA in the genu of the CC/forceps minor, bilat ACR, bilat post limb IC (CST) with delay aversion
Bos et al., 2017	35	11.2 (2.6)	26	105.5 (15.9)	NA	4	8	36	12.1 (2.2)	29	109.6 (16.4)	TBSS + network	No group differences
Bouziane et al., 2018 (children)	49	11.34 (0.87)	49	104.62 (18.08)	ADHD-C: 21 ADHD-I: 27 ADHD-HI: 1	3	49	11	11.36 (0.84)	11	121.6 (10.9)	TBSS + ROI	No group differences
Bu et al., 2020	38	8.79 (2.11)	29	120.63 (16.34)	NA	0	38	34	9.29 (1.67)	21	122.18 (13.58)	TG	Reduced FA and increased MD in the cerebral peduncle; increased FA in the R CST. ASSOC: FA in the CST and RD in the R peduncle with attention
Cao et al., 2010	28	13.3 (1.5)	28	102.8 (12.9)	ADHD-C: 12 ADHD-I: 16	9	23	27	13.2 (0.9)	27	115.1 (12.7)	ROI	Reduced FA in the isthmus of the CC
Cao et al., 2013	30	10.3 (1.9)	30	107.1 (14.4)	ADHD-C: 15 ADHD-I: 15	11	30	30	10.3 (1.6)	30	121.7 (14)	TG + network	Decreased global efficiency and increased shortest path length in the L fronto-parieto-occipital cortices. ASSOC: decreased structural connectivity in the prefrontal circuitry and increased connectivity in the orbitofrontal-striatal circuitry with inattention and HI symptoms respectively.
Çelik et al., 2020	13	16.0 (1.2)	13	NA	ADHD-C: 13	NA	NA	13	16.5 (1.3)	13	NA	TG + network	Increased structural connectivity in ADHD + cannabis use in a network including L hippocampus, amygdala, MTG, cerebellum and R thalamus. Other edges linked the L amygdala with insula and IFG, and another the cerebellar lobules.
Cha et al., 2015	30	9.4 (2.0)	24	99.1 (15.9)	ADHD-C: 24 ADHD-I: 5 ADHD-HI:1	16	30	31	10.5 (2.1)	21	109.1 (15.7)	TG	Reduced fronto-accumbal connectivity. ASSOC: fronto-accumbal WM and aggression
Chen et al., 2015	33	9.7 (2.2)	32	104.2 (5.2)	ADHD-C: 33	0	33	35	10.5 (1.8)	33	107.1 (6.4)	VBA	Increased FA in the L posterior CING . ASSOC: FA in the CING with behavioral measures and executive functions
Chiang et al., 2015	50	11.26 (2.93)	38	110.28 (11.52)	ADHD-C: 26 ADHD-I: 24	22	NA	50	11.22 (2.79)	38	111.78 (11.02)	TG	Reduced GFA in the L frontostriatal tracts, bilat SLF and R CING. ASSOC: R SLF GFA and inattentive symptoms
Chiang et al., 2016	45	11.36 (2.86)	33	109.98 (11.6)	ADHD-C: 22 ADHD-I: 22 ADHD-HI: 1	0	35	45	11.29 (2.71)	33	111.42 (11.21)	TG	Reduced GFA in the L FS tract, SLF, arcuate and R CING. ASSOC: FA in the FS, SLF and arcuate with executive functions
Chiang et al., 2020	50	12.13 (2.33)	42	103.70 (13.22)	NA	0	50	50	12.00 (2.39)	42	109.35 (11.42)	TG	Increased AD in the perpendicular fasciculus, SLF I, CST, and CC. ASSOC: AD in the SLF I, CST and CC and symptom severity, sustained attention and working memory
Choi et al., 2008	1) ADHD: 15 2) ADHD + tic/Tourette: 24	1) 9.6 (1.8). 2) 10.4 (1.1)	1) 15 2) 24	1) 106.5 (11) 2) 102.7 (10.9)	1) ADHD-C: 15 2) ADHD-C: 24	1) 5 2) 24	1) 0 2) 1	9	9.7 (1.7)	9	121.1 (8.8)	VBA	Decreased FA in the L MCP and R frontal lobe, increased FA in the R middle occipital WM
Chuang et al., 2013	12	14.8 (1.4)	12	99.4 (11.7)	ADHD-C: 8 ADHD-I: 4	7	0	14	15.7 (NA)	14	102.4 (9.3)	TBSS	Lower FA in the MCP, CST, L ILF, IC, L OR, CC (splenium), L ACR, PCR. ASSOC: MCP FA with global cognitive performance; IC FA with executive functions; and PCR FA with attention
Cooper et al., 2014	17	15.6 (1.3)	17	87.6 (9.8)	ADHD-C: 17	NA	1	17	16.9 (1.2)	17	106.9 (7.6)	TBSS	No group differences. ASSOC: FA and RD in the R posterior limb of the IC/CST, R cerebral peduncle and midbrain with autistic traits
Cooper et al., 2015	17	15.6 (1.3)	17	87.6 (9.8)	ADHD-C: 17	NA	1	17	16.9 (1.2)	17	106.9 (7.6)	TG	No group differences. ASSOC: FA and RD in the L subgenual CING with symptom severity, in the R CST with autistic traits
Damatac et al., 2020	258	17.4 (3.6)	182	95.1 (16)	NA	90	56	322	17.2 (3.7)	152	104.3 (14.9)	TG	No group differences. ASSOC: FA in the R CING and HI symptom severity
Davenport et al., 2010	14	15.0 (2.34)	12	113.1 (15.7)	ADHD-C: 13 ADHD-I: 1	NA	0	26	14.8 (2.41)	16	114.2 (10.4)	VBA	Increased FA in L inferior and R superior frontal regions. Reduced FA in the L fornix
de Luis-Garcia et al., 2015	1)medication naïve: 16 2) treated: 24	1) 7.62 (1.36) 2) 8.50 (1.1)	1) 16 2) 24	1) 100.3 (18.2) 2) 103.3 (13.3)	NA	0	16	26	8.23 (1.53)	26	120.3 (14.5)	TBSS + TG	No case-control differences. Medication-naïve vs treated subjects and controls: reduced MD in the ILF, UNC and CC. ASSOC: MD in CST and attention performance
De Zeeuw et al., 2012a	30	9.6 (2.3)	27	104 (17)	ADHD-C:19 ADHD-I: 6 ADHD-HI: 5	11	NA	34	10.2 (2.3)	30	111 (16)	TG	Reduced frontostriatal FA
de Zeeuw et al., 2012b	30	9.6 (2.3)	27	104 (17)	ADHD-C:19 ADHD-I: 6 ADHD-HI: 5	11	NA	34	10.2 (2.3)	30	111 (16)	VBA	No group differences
Douglas et al., 2018	1) whole ADHD sample: 56 2) medication naïve: 29	1) 12.6 (3.2) 2) 12.6 (3.8)	1) 39 2) 21	1) 106.2 (13.1) 2) 106.1 (13.9)	NA	1) 27 2) 0	29	17	13.2 (2)	6	110.4 (13.1)	TG	Differences in diffusivity asimmetry indices in the CING, ILF, SLF and CST
Ercan et al., 2016	1) ADHD-C: 24 2) ADHD-I: 24 3) ADHD-RI: 24	1) 10.5 (1.7) 2) 11.1 (2.0) 3) 10.7 (2.2)	1) 24 2) 18 3) 18	1) 112.5 (20.2) 2) 106.8 (16.6) 3) 108.0 (19.1)	1) ADHD-C: 24 2) ADHD-I (I): 24 3) ADHD-I (RI): 24	1) 8 2) 3	1) 24 2) 24 3) 24	24	10.8 (1.3)	18	111.4 (17.8)	TBSS	No case-control differences. ADHD-C vs ADHD-I: increased RD (bilat) and AD (mostly L) including splenium and body of the CC, IC, superior, anterior and posterior CR, PTR, SLF. ADHD-I vs ADHD-RI: increased FA in the bilat PCR, R superior CR, L CING
Fall et al., 2015	11	9.8 (1.7)	10	VIQ: 112.5 (14.4) PIQ: 103.1 (14.4)	ADHD-C: 11	0	11	11	10.8 (1.7)	8	VIQ: 122.4 (11.7) PIQ: 111.9 (8.8)	ROI	No group differences. ASSOC: MD in the bilat caudate, putamen and thalamus with RT and RTV
Fayed et al., 2007	22	9 (2.91)	18	NA	NA	0	22	8	7.5 (3)	4	NA	ROI	No group differences
Francx et al., 2015	1) Remitters: 42 2) Persisters: 59	1) T1: 11.9 (2.4) T2: 17.9 (2.5) 2) T1: 11.9 (2.8) T2: 17.8 (2.7)	1) 36 2) 45	1) T1: 94.5 (12.8) T2: 96.3 (13.2) 2) T1: 95.9 (13.9) T2: 94.1 (15.1)	1) 42 2) 59	1) 13 2) 30	NA	40	T1: 12.9 (2.3) T2: 17.8 (2.1)	10	T1: 106.4 (13.4) T2: 106.7 (13.5)	TBSS + TG	ASSOC: lower FA and higher MD in the left CST and SLF, and higher MD in CC and CING, with age-related improvement of HI symptoms
Francx et al., 2016	129	17.8 (3.2)	90	96.9 (15.6)	ADHD-C: 129	41	NA	204	17.3 (3.5)	84	103.0 (13.1)	TBSS	Increased FA in the forceps major. Reduced FA in the IC, CC and PostCG. Increased MD in the PostCG and reduced MD in the thalamus. Increased MA in the bilat superior CR
Fuelscher et al., 2021	76	[9.4-11.5]	56	NA	NA	NA	59	68	[9.6-11.9]	38	NA	TG + FBA	Reduced FD in the bilat CST, frontopontine tract, and L IFOF. ASSOC: FD in the left frontopontine tract and symptom severity
Gau et al., 2015	32	11.4 (2.3)	29	109 (12.2)	ADHD-C: 19 ADHD-I: 13	NA	15	32	NA	NA	112.4 (10.0)	TG	Reduced GFA in four fronto-striatal tracts:caudate–dorsolateral, caudate–medial prefrontal, caudate–orbitofrontal and caudate–ventrolateral tracts. ASSOC: FA and school dysfunction, mediated by executive functions and ADHD symptom severity
Graziano et al., 2022	102	NA (young children)	NA	NA	NA	70	NA	96	NA	NA	NA	TG	Reduced FA in the bilat UNC, IFOF and CST and L ILF. ASSOC: FA in the bilat UNC and L IFOF and callous-unemotional behaviors
Griffiths et al., 2021	37	13.31 (2.53)	NA	NA	ADHD-C:20 ADHD-I:17	NA	NA	26	13.24 (2.87)	NA	NA	TG + network	Reduced local efficiency in the L pallidum, putamen, MTG, PostCG and transverse temproal area; bilat thalamus, and R caudate, pars opercularis and amygdala. ASSOC: local effeciency in the R thalamus, isthmus, CING and pars triangularis, FG, SMG, MFG and in the L SFG and bilat PreCG with treatment-related symptom improvement
Hamilton et al., 2008	17	11.95 (2.32)	17	92.25 (14.55)	ADHD-C:1 ADHD-I: 5 ADHD-HI: 9	10	NA	16	11.72 (2.48)	16	103.35 (10.09)	ROI	Reduced FA in the CST and SLF
Helpern et al., 2011	12	14.4 (1.6)	9	105 (18)	ADHD-C:7 ADHD-I: 5	2	4	13	14.8 (1.7)	7	110 (16)	ROI	No group differences in WM microstructural complexity but lack of age-related changes observed in controls
Hong et al., 2014	1) whole ADHD sample: 71 2) ADHD-C: 39 3) ADHD-I: 26	1) 9.39 (2.59) 2) 9.30 (2.47) 3) 9.78 (2.81)	1) 15 2) 6 2) 6	1) 106.06 (12.47) 2) 105.38 (12.71) 3) 108.31 (11.87)	ADHD-C: 39 ADHD-I: 26 ADHD-HI:1 NOS: 5	16	61	26	10.04 (2.47)	13	117.27 (10.39)	TG + network	Reduced connectivity in a network spanning frontal, striatal, and cerebellar regions. ADHD-C vs ADHD-I: decreased connecitivity in network including SFG, CING, and SMA. ASSOC: FA and omissions, commissions and RTV
Hyde et al., 2021a	50	10.38 (0.43)	35	96.28 (13.34)	NA	26	50	56	10.49 (0.45)	31	102.73 (13.36)	FBA	Reduced FD, FC and FDC in the R CST and reduced FDC in the L CST
Hyde et al., 2021b	55	9.6 (0.42)	39	96.18 (13.16)	NA	32	55	61	9.58. (0.45)	35	103.41 (13.25)	TG	ASSOC: AFD in the R SLF I, and volume of R SLF I, II and III with fine motor control
Jacobson et al., 2015	60	9.9 (1.3)	30	108.6 (12.3)	ADHD-C: 41 ADHD-I: 17 ADHD-HI: 2	19	NA	60	10.2 (1.1)	30	111.9 (10.0)	ROI	ADHD boys: reduced FA in bilat M1; ADHD girls: increased FA bilat within medial OFC. ASSOC: FA and commissions and RTV
King et al., 2015	19	12.68 (2.14)	10	107.53 (11.49)	ADHD-C: 10 ADHD-I: 9	3	3	24	14.42 (2.76)	12	113.59 (8.40)	TBSS + ROI	Reduced FA in the bilat SLF, forceps major, L CING, and bilat CC. Females vs males: increased FA in the CST, ILF and SLF
Kobel et al., 2010	14	10.43 (1.34)	14	NA	NA	7	0	12	10.92 (1.62)	12	NA	VBA	Reduced FA in the L ACR and R MCP; reduced FA in the L temporo-occipital WM
Langevin et al., 2014	1) ADHD only: 23 2) ADHD +DCD:19	1) 11.78 (2.99) 2) 11.39 (2.89)	1) 20 2) 19	1) 107.67 (13.07); 2) 102.35 (14.06)	NA	2) 19	NA	26	11.58 (3.18)	14	114.12 (13.07)	TG	Reduced FA in the anterior/superior frontal CC. ASSOC: FA in the CC with attention/executive functions. FA in the bilat SLF and motor control in those with comorbid DCD
Lawrence et al., 2013	56	12.6 (3.2)	39	105.3 (13.1)	NA	40	29	17	13.2 (2.0)	6	110.4 (13.3)	TG	Increased MD and AD in the ATR, forceps minor and SLF
Lee et al., 2009	11	10.45	7	NA	NA	NA	11	9	10.56	6	NA	ROI	Reduced FA in L ILF and IFOF
Lee et al., 2021	1) ADHD-C: 70 2) ADHD-I: 75	1) 10.0 (2.4) 2) 11.5 (2.9)	1) 56 2) 54	1) 75.8 (41.3) 2) 84.6 (36.2)	1) ADHD-C: 70 2) ADHD-I: 75	1) 19 2) 20	NA	56	10.2 (2.6)	20	66.3 (53.8)	TG + network	Reduced structural connectivity and network efficiency in the DMN
Lei et al., 2014	1) ADHD-C: 28 2) ADHD-I: 28	9.3 (1.3)	25	1) 99.6 (11.3) 2) 96.1 (12.1)	1) ADHD-C: 28 2) ADHD-I: 28	0	1) 28 2) 28	28	9.2 (1.4)	25	99.1 (10.3)	VBA	ADHD-C vs controls: decreased AD and RD in the L MFG, SMA and precuneus; reduced FA in the L PHG, and decreased AD in the L precuneus and R CING. Increased RD in the R FG and MTG, and in the L cuneus, lingual gyrus and STG. ADHD-C vs ADHD-I: metric differences in the R thalamus and caudate, L PostCG, and SMA
Li et al., 2010	24	9.62 (2.19)	22	NA	ADHD-C: 18 ADHD-I: 6	8	24	20	10.12 (1.83)	18	NA	VBA	Increased FA in the R frontal WM. ASSOC: FA with executive functions
Lin et al., 2014	28	11.54 (2.30)	25	107.86 (14.75)	ADHD-C: 17 ADHD-I: 11	0	11	28	11.57 (2.75)	25	107.71 (12)	TG	Reduced GFA in four bilateral frontostriatal tracts. ASSOC: GFA in the CING and frontostriatal tracts with RTV
Lin et al., 2020	14	7.9 (2.1)	0	120.36 (17.21)	ADHD-C: 6 ADHD-I: 7 ADHD-HI: 1	0	14	10	8.9 (0.6)	0	121.60 (18.17)	TG	ADHD girls: reduced FA and increased RD in the callosal forceps major. ASSOC: FA with HI symptoms and impulsivity, RD with impulsivity
Lin et al., 2022	1) whole ADHD sample: 76 2) males: 53 3) females: 23	1) 8.80 (2.25) 2) 9.04 (2.22) 3) 8.26 (2.26)	53	1) 119.78 (15.10) 2) 119.98 (14.83) 3) 119.30 (16.02)	NA	0	1) 76 2) 53 3) 23	1) 37 2) 20 3) 17	1) 9.51 (1.68) 2) 9.20 (1.83) 3) 9.88 (1.45)	20	1) 121.14 (13.37) 2) 121.30 (120.05) 3) 120.94 (15.15)	TG	Reduced FA in the occipital and superior parietal callosal tracts; increased MD in the posterior, superior parietal and anterior frontal tracts. ADHD girls: decreased FA and volume of the occipital tract and increased MD in the posterior and superior parietal tracts. ADHD boys: decreased volume of the frontal tract.
Malisza et al., 2012	20	11.99 (1.32)	18	96.55 (16.87)	NA	5	5	21	12.6 (1.29)	16	107.81 (13.08)	VBA	No group differences
Mazzetti et al., 2022	22	10.7 (1)	100	NA	NA	NA	0	26	10.7 (1.3)	100	NA	TG	ASSOC: FA in the ATR and symptom severity. Dorso-ventral gradient in SLF FA and methylphenidate-induced behavioral improvement
Nagel et al., 2011	20	8.05 (0.69)	13	106.5 (12.8)	ADHD-C: 11 ADHD-I: 8 ADHD-HI: 1	10	17	16	8.31 (0.70)	4	115.4 (12.9)	TBSS	Reduced FA in fronto-parietal, fronto-limbic, cerebellar, CR and temporo-occipital WM. Lower MD in the posterior limb of the IC and fronto-parietal WM, and greater MD in fronto-limbic WM
O’Conaill et al., 2015	19	11.93 (1.33)	17	95.79 (16.97)	NA	5	4	21	12.60 (1.29)	16	107.81 (13.08)	TBSS	No group differences
O’Neil et al., 2019	1) ADHD + PAE: 9 2) ADHD: 11 3) ADHD/?PAE: 4	1) 12.4 (2.1) 2) 11.7 (2.4) 3) 11.8 (1.4)	1) 8 2) 9 3) 1	1) 88.1 (18.0) 2) 92.8 (14.1) 3) 81 (20.3)	1)ADHD-C: 6 ADHD-I: 3 2)ADHD-C: 5 ADHD-I: 5 NOS: 1 3) ADHD-C: 2 NOS: 2	1) 10 2) 11 3) 6	1) 7 2) 10 3) 0	9	13.1(2.2)	2	92.0 (15.4)	TBSS	No group differences
O’Neil et al., 2022	1) ADHD + PAE: 23 2) ADHD - PAE: 19	1) 9.7 (1.6) 2) 10.7 (0.9)	1) 16 2) 12	1) 94.9 (12.8) 2) 107.5 (11.5)	NA	NA	NA	28	11.3 (1.6)	13	116.7 (15.6)	ROI	AD and MD paired with spectroscopic measures of supraventricular WM were discriminative between ADHD (whole group) and controls
Park et al., 2016	1) ADHD+PTE: 29 2) ADHD-PET: 25	1) 9.20 (2.89) 2) 9.12 (1.97)	1) 26 2) 20	1) 104.86 (11.90) 2) 106.42 (13.40)	NA	NA	NA	1) TDC+PTE: 18 2) TDC-PTE: 23	1) 9.78 (3.19) 2) 8.96 (2.01)	1) 12 2) 11	1) 111.11 (15.03) 2) 113.18 (10.99)	TBSS	Widespread areas of increased FA and reduced MD
Pastura et al., 2016	13	8 (1.2)	10	105 (11.5)	ADHD-C: 8 ADHD-I: 4 ADHD-HI: 1	8	1	14	9 (1.3)	10	106 (17.7)	TBSS	Lower FA in the splenium of the CC, R SLF, bilat retrolenticular IC and IFOF, L EC and PTR
Pavuluri et al., 2009	13	13.4 (3.0)	12	94.0 (13.7)	NA	NA	NA	15	13.7 (2.7)	6	113.7 (13.5)	ROI	Reduced FA in the ACR, anterior limb of the IC and the superior IC. Increased ADC in the ACR, anterior and posterior limbs of the IC, superior IC, CING, ILF, and SLF
Peterson et al., 2011	16	11.28 (1.55)	11	108 (14.03)	ADHD-C: 9 ADHD-I: 7	6	NA	16	11.15 (2.14)	11	115 (10.28)	VBA + ROI	VBA: increased FA in the R SFG and PTR, and L CING, lingual gyrus, and PHG. ROI: increased FA in the L sagittal stratum. ASSOC: FA in the L sagittal stratum and symptom severity
Qian et al., 2021	34	9.44 (1.67)	32	104.09 (15.56)	NA	NA	NA	30	9.67 (2.88)	22	107.07 (18.43)	TG + network	Increased betweenness centrality in the L hyppocampus. ASSOC: betweenness centrality in the R hyppocampus and oppositional behavior
Qiu et al., 2011	15	12.65 (1.82)	15	NA	ADHD-I: 15	NA	NA	15	13.21 (1.73)	15	NA	VBA	Decreased FA in the forceps minor, IC, CR, splenium of the CC, and bilat basal ganglia
Ray et al., 2014	20	[8-12]	NA	NA	ADHD-C: 11 ADHD-I: 8 ADHD-HI: 1	NA	NA	20	[8-12]	NA	NA	TG + network	Under-connectedness inside rich-club organization with reduced GFA
Rossi et al., 2015	29	10.14 (1.94)	20	110.60 (14.94)	ADHD-I: 29	NA	NA	29	10.10 (1.63)	19	120.21 (14.96)	TBSS	No group differences. ASSOC: FA in the bilat CST and UNC, R CING and IFOF, forceps minor and major with attention performance
Saad et al., 2021	1) ADHD-C: 19 2) ADHD-I: 18	1) 13.25 (2.53) 2) 13.35 (2.65)	1) 15 2) 14	NA	1) ADHD-C: 19 2) ADHD-I: 18	1) 7 2) 3	1) 8 2) 12	28	13.09 (2.63)	9	NA	TBSS + TG + network	No group differences in TBSS/TG analysis. Difference in nodal degree (R insula)
Saenz et al., 2020	36	10.47 (1.33)	21	105.53 (10.85)	ADHD-C: 36	0	NA	20	118.10 (13.26)	10	109.39 (9.22)	TBSS	No group differences. ASSOC: MD in the SLF and CING with working memory
Shang et al., 2013	25	11.4 (2.1)	22	108.4 (12.7)	ADHD-C: 16 ADHD-I: 8 ADHD-HI: 1	0	7	25	11.4 (2.7)	22	111.1 (10.9)	TG	Reduced GFA in four fronto-striatal tracts. ASSOC: GFA and symptom severity and executive functions
Silk et al., 2009a	15	12.6 (2.4)	15	PIQ 104.9 (11.5)	ADHD-C: 15	0	12	15	12.9 (2.6)	15	PIQ 111.6 (9.2)	ROI	No group differences in tract metrics
Silk et al., 2009b	15	12.6 (2.4)	15	PIQ 104.9 (11.5)	ADHD-C: 15	0	12	15	12.9 (2.6)	15	PIQ 111.6 (9.2)	TBSS	Increased FA in the R CING and SLF, and in the L UNC and ILF.
Silk et al., 2016	21	13.3 (1.8)	21	94.3 (11.3)	ADHD-C: 21	NA	15	22	14.6 (2.2)	22	110.3 (8.5)	TG	L lateralized FA in the putamen-ventrolateral PFC. ASSOC: FA and symptom severity
Stephens et al., 2021	1) ADHD: 62 2) ADHD+ASD: 16	1) 10.3 (0.5) 2) 10.7 (0.5)	1) 45 2) 12	NA	NA	NA	NA	73	10.4 (0.4)	41	NA	TG	Reduced FA in R CING. ASSOC: FA in the L CING and emotional problems in comorbid ADHD+ASD
Sun et al., 2018	83	10.83 (2.30)	71	NA	ADHD-C: 43 ADHD-I: 40	NA	83	87	11.21 (2.51)	72	NA	ROI + machine learning	Features discriminating ADHD vs controls included FA in the L cerebral peduncle; ADHD-C vs ADHD-I included FA in the L EC
Svatkova et al., 2016	1) ADHD-C: 13 2) ADHD-PI: 20	1) 12.78 (2.32) 2) 14.95 (2.37)	1) 10 2) 16	1) 109.5 (7.6) 2) 103.9 (11.2)	1)ADHD-C: 13 2) ADHD-I: 20	0	1) 6 2) 6	23	14.15 (2.84)	16	110.4 (9.2)	TBSS	ADHD-I vs controls: higher FA in the ATR, bilat ILF and L CST. ADHD-C vs controls: higher FA in the bilat CING. ADHD-I vs ADHD-C: higher RD in the forceps minor. ASSOC: FA in the R ILF and CING with executive functions; FA in the R ILF with attentive symptom severity
Tamm et al., 2012	12	15.8 (1.5)	12	106.1 (10.8)	ADHD-C: 12	11	NA	12	15.6 (0.8)	12	111.6 (11.7)	TBSS	Increased FA and AD in ACR, ATR, UNC, IFOF, forceps; higher AD in the genu of the CC.
Tremblay et al., 2020	60	10.6 (2.6)	50	101	ADHD-C: 43 ADHD-I: 13 ADHD-HI: 4	NA	NA	16	10.5 (4.86)	5	111	TG	No significant group differences
Tsai et al., 2021	77	11.77 (2.41)	62	108.4 (10.6)	NA	11	60	105	12.39 (2.82)	85	110.3 (11.6)	TG	ASSOC: GFA in widespread tracts including bilat ILF, stria terminalis, arcuate; L CING, IFOF, UNC, TR, posterior commissure, CC; and R SLF II with emotion dysregulation, symptom severity and IQ
Unsel-Bolat et al., 2020	1) SCT+ADHD-IA: 24 2) ADHD-IA: 57	1) 9.25 (1.3) 2) 10.8 (1.9)	1) 17 2) 36	NA	1) ADHD-I: 24 2) ADHD-I: 57	0	1) 24 2) 57	24	10.8 (1.3)	18	NA	TBSS	ADHD+ SCT symptoms: higher FA in the bilat anterior and posterior limbs of the IC, cerebral peduncle and fornix
Wang et al., 2008	16	11.4 (1.2)	12	NA	ADHD-C: 4 ADHD-I: 12	NA	16	16	10.9 (1.4)	11	NA	TG	Reduced FA in R pericallosal WM and bilat CING. ASSOC: FA in R CING and bilat MCP and reaction time
Wang et al., 2020	1) Dataset 1: 25 2) Dataset 2: 11	1) 11.05 (1.68). 2) 11.71 (2.13)	1) 24 2) 11	1) 108.1 (16.7) 2) 114.36 (13.86)	1) ADHD-C: 25 2) ADHD-C: 11	NA	NA	1) 45 2) 26	1) 11 (1.40) 2) 11.98 (1.77)	1) 35 2) 26	1) 121.3 (13.6) 2) 119.04 (12.80)	TG + network	Increased trace-map distance in regions near the L parietooccipital junction
Wu et al., 2014	25	11.36 (2.14)	22	108.40 (12.69)	ADHD-C: 16 ADHD-I: 8 ADHD-HI: 1	19	7	25	11.40 (2.69)	22	111.12 (10.91)	TG	Reduced GFA in four fronto-striatal tracts. ASSOC: GFA in the L orbito-frontal caudate tract and inattentive symptom severity
Wu et al., 2017	83	11 (1.99)	73	107.1 (14.41)	NA	32	69	122	10.6 (1.81)	60	118.7 (13.2)	TBSS	Decreased FA and increased RD mainly in CC, L SLF, L anterior and superior CR. ASSOC: reduced FA and inhibition; increased FA and HI symptom severity
Wu et al., 2019	80	10.95 (1.95)	71	106.96 (14.37)	NA	32	67	119	10.62 (1.82)	58	118.64 (13.16)	TBSS + multimodal	Reduced FA, increased RD and MD in the ATR, CING, body of the CC and CST. ASSOC: FA in bilat SLF and IFOF, and RD and MD in CST and ATR with symptom severity
Wu et al., 2020a	30	10.64 (1.69)	23	95 (13.81)	NA	NA	8	28	10.61 (1.73)	18	112 (17.51)	TG	Lower axonal/cellular packing density and volume primarily in the R SLF-II, thalamus to PreCG and SFG, caudate to OFC and PreCG, thalamus to L paracentral gyrus and MFG, and bilat CING
Wu et al., 2020b	83	11 (1.99)	73	107.1 (14.41)	NA	NA	NA	122	10.6 (1.81)	60	118.7 (13.2)	TBSS	FA lateralization index increased in PTR. ASSOC: lateralization index in EC and inattentive symptoms
Wu et al., 2022	1) ADHD-C: 41 2) ADHD-I: 76	1) 8.44 (1.21) 2) 8.92 (1.47)	1) 36 2) 59	NA	1) ADHD-C: 41 2) ADHD-I: 76	0	100	83	9.39 (1.37)	44	NA	TBSS	ADHD-C vs controls: increased volume body of the CC. ADHD-I vs controls: increased AD in the CC, bilat CR, EC, SLF, and L IC, CING, CST, and superior fronto-occipital fasciculus. ADHD-C vs ADHD-I: reduced AD in the CC, bilat EC, IC, CR; L TR, tapetum and superior fronto-occipital fasciculus; and R SLF
Xia et al., 2012	19	10.9 (2.3)	14	102.8 (17)	ADHD-C: 19	NA	11	19	12.2 (2.3)	10	110.5 (12.6)	TG	Decreased FA and volume in the tracts between thalamus and striatum, hippocampus, and PFC
Yoncheva et al., 2016 (children)	82	10.63 (2.8)	64	105.52 (14.9)	ADHD-C: 50 ADHD-I: 26 ADHD-HI: 4	27	59	80	11.04 (2.6)	55	108.98 (14.3)	TBSS	Reduced global MA. ASSOC: MA and symptom severity
Yoo et al., 2020	1) Training sample: 47 2) Independent sample: 18	1) 10.06 (2.24) 2) 9.44 (2.41)	1) 37 2) 12	1) 110.53 (14.38) 2) 114.17 (13.08)	1) ADHD-C: 14 ADHD-I: 22 ADHD-HI: 3 NOS: 8 2) ADHD-C: 5 ADHD-I: 5 ADHD-HI: 4 NOS: 4	0	NA	1) 47 2) 18	1) 10 (2.60) 2) 10.06 (2.69)	1) 29 2) 10	1) 110.53 (14.38) 2) 114.61 (14.01)	TBSS + machine learning	Overall diffusivity scalars contributed to classification ADHD vs controls
Zhan et al., 2017	32	11.1 (0.3)	25	104.3 (2.4)	ADHD-C: 32	0	23	32	11.9 (0.4)	19	108.1 (2.3)	ROI + network	Increased RD in subgenual CING and premotor cortex. ASSOC: RD and symptom severity
Zhou et al., 2021	116	[9-10]	71	NA	NA	0	NA	116	[9-10]	71	NA	ROI + machine learning	Discriminative features included: FA in the R pars orbitalis and L PostCG; MD in the R CING and L amygdala; AD in the R CING and thalamus

Clinicodemographic characteristics, imaging analysis method, and main findings are reported. References are listed in Supplementary material.

*ACR* anterior corona radiata, *AD* axial diffusivity, *ADC* apparent diffusion coefficient, *ADHD* Attention-deficit/hyperactivity disorder, *ADHD-C* ADHD combined presentation, *ADHD-HI* hyperactive/impulsive presentation, *ADHD-I* ADHD inattentive presentation, *AFD* apparent fiber density, *ASD* autism spectrum disorder, *ASSOC* association, *ATR* anterior thalamic radiation, *Bilat* bilateral, *CC* corpus callosum, *CING* cingulum, *CR* corona radiata, *CST* corticospinal tract, *DCD* developmental coordination disorder, *DMN* default-mode network, *EC* external capsule, *FA* fractional anisotropy, *FC* fiber cross-section, *FD* fiber density, *FDC* fiber density/cross-section, *FG* fusiform gyrus, *FS* frontostriatal tract, *GFA* generalized fractional anisotropy, *GM* gray matter, *HI* hyperactive/impulsive, *IC* internal capsule, *IFG* inferior frontal gyrus, *IFOF* inferior fronto-occipital fasciculus, *ILF* inferior longitudinal fasciculus, *IQ* intelligent quotient, *L* left, *M1* primary motor cortex, *MA* mode of anisotropy, *MCP* middle cerebellar peduncle, *MD* mean diffusivity, *MFG* middle frontal gyrus, *MTG* middle temporal gyrus, *NA* not available, *NOS* not otherwise specified, *OFC* orbitofrontal cortex, *OR* optic radiation, *PCR* posterior corona radiata, *PFC* prefrontal cortex, *PHG* parahippocampal gyrus, *PostCG* postcentral gyrus, *PreCG* precentral gyrus, *PTR* posterior thalamic radiation, *R* right, *RD* radial diffusivity, *ROI* region of interest, *RT* reaction time, *RTV* reaction time variability, *SCT* sluggish cognitive tempo, *SFG* superior frontal gyrus, *SLF* superior longitudinal fasciculus, *SMA* supplementary motor area, *SMG* supramarginal gyrus, *STG* superior temporal gyrus, *TBSS* tract-based spatial statistics, *TG* tractography, *TR* thalamic radiation, *UNC* uncinate, *VBA* voxel-based analysis, *WM* white matter.

**Table 2:** Diffusion imaging studies in adults or mixed pediatric/adult samples with ADHD.

STUDY	ADHD participants							Control participants				Imaging method	Main findings
	*N*	Age	Males (*N*)	IQ	ADHD presentation (*N*)	Comorbidities (*N*)	Drug naïve (*N*)	*N*	Age	Males (*N*)	IQ		(ADHD vs controls and associations)
Bode et al., 2015	30	22.59 (0.76)	21	98.17 (18.22)	NA	10	30	30	23.09 (0.64)	22	110.00 (22.48)	TBSS	Increased FA and reduced RD in L forceps minor; reduced AD in the forceps minor and genu of the CC
Bouziane et al., 2018 (adults)	48	28.59 (4.64)	48	107.86 (7.5)	ADHD-C: 32 ADHD-I: 16	0	48	12	25.18 (1.86)	12	108.08 (5.52)	TBSS + ROI	Decreased FA in the bilat SLF, CC and ATR
Chaim et al., 2014	22	28.8 (4.9)	14	NA	ADHD-C: 10 ADHD-I: 12	7	22	19	28.7 (5.4)	12	NA	VBA	Increased FA in the bilat SFG, R MFG, L PostCG, bilat CING, bilat MTG and R STG. Reduced trace in the R SFG and bilat MFG, R PreCG, L MOG and bilat CING, as well as the L body and R splenium of the CC, R superior CR, R SLF and R IFOF
Chaim-Avancini et al., 2017	1) whole ADHD sample: 67 2) matched sample: 58 3) males: 52	1) 27 (6) 2) 26.9 (5.4) 3) 27 (5.1)	1) 52 2) 44 3) 44	NA	1) ADHD-C: 31 ADHD-I: 36 2) ADHD-C: 28 ADHD-I: 30 3) ADHD-C: 27 ADHD-I: 25	12	1) 67 2) 58 3) 52	2) matched sample: 58 3) males: 44	2) 26.7 (5.7) 3) 27 (5.5)	2) 44 3) 44	NA	ROI + machine learning	FA and trace was discriminative between ADHD and controls in several bilat WM regions: CST, ILF and SLF, IFOF, UNC, CC, fornix, CING, ATR, superior CR, MCP, and brain stem
Chiang et al., 2017	32	23.35 (3.34)	32	109.69 (10.58)	NA	0	18	29	22.4 (3.3)	18	114.59 (11.06)	TG	No group differences
Chiang et al., 2022	64	28.70 (7.85)	35	117.71 (13.12)	NA	NA	64	81	28.39 (7.90)	45	114.58 (10.61)	TG	Increased GFA in the L FAT, R ILF, and L perpendicular fasciculus. Reduced GFA in the R SLF I, L SLF II, R FS, R medial lemniscus, R inferior TR and callosal fibers. ASSOC: GFA in the R SLF I and HI symptoms
Cortese et al., 2013	1) whole ADHD sample: 51 2) persisters: 15 3) remitters: 25	1) 41.3 (2.8) 2) 41.8 (3) 3) 41.3 (2.6)	1) 51 2) 15 3) 25	1) 101.3 (13.7) 2) 99.3 (13) 3) 103.8 (13.1)	ADHD-C: 3 ADHD-I: 6 ADHD-HI: 6	1) 11 2) 4 3) 5	4	66	42.2 (3.1)	66	111.1 (14.3)	TBSS	Reduced FA in the R superior and posterior CR, R SLF, L posterior TR, retrolenticular part of the IC, and sagittal stratum. No difference between ADHD persisters and remitters
Dramsdahl et al., 2012	29	32.9 (7.1)	15	110.6 (14.3)	ADHD-C:19 ADHD-I: 7 ADHD-HI: 3	NA	13	37	30 (6.4)	14	116.7 (9.2)	TBSS	Reduced FA in the isthmus/splenium of the CC
Elliott et al., 2021	74	NA (adolescents/ young adults)	50	106.68 (13.22)	NA	NA	27	81	NA	36	114.67 (11.34)	TG	Increased structural connectivity between substantia nigra/ventral tegmental area and the limbic striatum, weaker connectivity with the executive striatum. ASSOC: tract integrity and impulsivity
Gehricke et al., 2017	32	25.31 (5.22)	26	NA	ADHD-C: 18 ADHD-I: 12 ADHD-HI: 2	10	NA	40	23.93 (3.60)	33	NA	VBA	Increased FA in the L EC and bilat OR. Decresead FA in the R STG, bilat MTG, R PostCG, CING, CC, bilat temporal stem, and R midbrain. Increased RD in the bilat PostCG, L MTG, CING, R IC, and R midbrain. Reduced RD in the L supraventricular WM and L pons. Increased MD in the L MTG, R IC, R midbrain, and L pons. Reduced MD in the CC and L pons. Increased AD in the R cuneus and R MOG. Reduced AD in the R PreCG, occipital lobe, and brainstem. ASSOC: multiple regions associated with chidhood symptoms (e.g. FA in the L subgyral WM of the frontal lobe and R putamen) and adult symptoms (e.g. FA in the R dentate, L CING, R lingual gyrus, L putamen, and R temporo-occipital gyrus)
Hearne et al., 2019	78	26.6 (5.5)	54	107.5 (10.4)	NA	0	78	118	25.8 (5.0)	76	109.8 (9.3)	TG + network	No group differences in structural connectivity
Kölle et al., 2022	53	27 (5.5)	38	113.2 (12.8)	NA	NA	53	50	26.2 (5.3)	32	116.2 (11.8)	VBA	Decreased FA in corticothalamic tract. ASSOC: FA with RTV
Konrad et al., 2010	37	32.5 (10.3)	21	109.8 (8.7)	ADHD-C: 37	0	37	34	30.2 (8.2)	16	111.4 (8.7)	VBA	Reduced FA and increased MD bilaterally in orbitomedial prefrontal WM (including IFOF, ATR, CC) and in the R anterior CING. Increased FA bilaterally in temporal WM (including IFOF and UNC). ASSOC: FA and MD in R SLF with attention. FA R UNC and R ATR and MD in the lingual gyrus with commissions
Konrad et al., 2012	37	32.5 (10.3)	21	109.8 (8.7)	ADHD-C: 37	0	37	34	30.2 (8.2)	16	111.4 (8.7)	ROI	Reduced FA in the L ILF and increased MD in the L IFOF. ASSOC: MD in the L ILF and attention
Li et al., 2019	40	32.1 (10.4)	21	NA	NA	NA	NA	53	32.5 (9.2)	28	NA	TG + network	Reduced asymmetric regional efficiency in the putamen, rolandic operculum and dorsal SFG. ASSOC: asymmetry scores in putamen, caudate, pallidum, PostCG, OLF and REC and symptom severity or cognitive performance
Li et al., 2021	40	32.1 (10.4)	21	NA	NA	NA	NA	51	32.4 (9.3)	27	NA	TG + network	Leftward asymmetry of PostCG, thalamus, and anterior CING in ADHD group only. ASSOC: asymmetry SFG with cognitive performance, and putamen with symptom severity
Luo et al., 2020a	1) whole ADHD sample: 32 2) remitters:16 3) persisters: 16	1) 24.66 (2.1) 2) 24.81 (2.3) 3) 24.39 (1.9)	1) 27 2) 13 3) 14	1) 96.81 (14.3) 2) 99.58 (14.2) 3) 94.11 (11.5)	1) 32 2) 16 3) 16	NA	0	35	24.24 (2.3)	30	104.21 (15)	TG	Decreased volume of the L parieto-insular fibers. Persisters vs remitters: decreased volume R hippocampo-frontal and R parieto-insular tracts and cortico-striatal tracts. ASSOC: FA of L caudate-parietal fibers and HI symptoms
Luo et al., 2020b	1) whole ADHD sample: 36 2) remitters:18 3) persisters: 18	1) 24.66 (2.0) 2) 24.79 (2.2) 3) 24.52 (2.0)	1) 30 2) 16 3) 14	1) 97.96 (14.1) 2) 99.22 (14.9) 3) 96.71 (13.6)	1) ADHD-C: 36	NA	NA	36	24.3 (2.3)	31	103.83 (15.4)	TG + multimodal machine learning	Fetures discriminative between ADHD (whole sample or persisters or remitters) and controls did not include diffusion imaging metrics.
Makris et al., 2008	12	41.3 (2.1)	7	NA	NA	NA	11	17	40.5 (2.1)	8	NA	ROI	Reduced FA in the R CING and SLF II
Ohta et al., 2020	55	31.2 (8.8)	42	106.3 (12.5)	NA	0	23	58	29.4 (6.7)	49	107.7 (7.7)	TBSS	Reduced FA and increased RD in the CC. ASSOC: RD and sensory sensitivity
Onnink et al., 2015	107	35 (10.30)	41	108.13 (14.43)	NA	10	20	109	36.08 (10.97)	47	110.97 (15.36)	TBSS	Reduced FA in CC, bilat CR, and TR. Higher MD and RD in overlapping regios, also encompassing IC and EC, saggital stratum, fornix, and SLF. ASSOC: FA and MD with impulsivity
Schweren et al., 2016	172	17.39 (3.05) [9-26]	116	96.62 (13.67)	ADHD-C: 66 ADHD-I: 82 ADHD-HI: 15	54	18	96	16.96 (3.26)	56	106.47 (14.09)	TG	Reduced FA in orbitofrontal-striatal tract
Shaw et al., 2015	1) persisters: 32 remitters: 43	1) 23.3 (3.7) 2) 24.1 (3.9)	1) 13 2) 26	1) 111 (12) 2) 114 (14)	NA	1) 9 2) 5	NA	74	24 (3.3)	44	113 (11)	tract-based analysis	ADHD persisters: reduced FA in the bilat UNC and R IFOF. No differences between remitters and controls. ASSOC: FA in the L IFOF and UNC; RD in the bilat IFOF, UNC and SLF; and AD in the R ILF with inattentive symptoms
Sidlauskaite et al., 2015	18	30.11 (9.78)	9	112.05 (13.99)	ADHD-C: 12 ADHD-I: 6	NA	NA	21	26.95 (8.52)	12	116.90 (11.24)	TG + network	Preserved global but altered local network organization. Affected nodes included superior occipital, supramarginal, superior temporal, inferior parietal, angular and IFG, as well as putamen, thalamus and posterior cerebellum. ASSOC: ADHD symptom severity
Tung et al., 2021	279	18.03 (8.3) [5–40]	205	106.6 (12)	NA	0	NA	626	20.16 (8.5)	376	110 (11)	TG	Widespread GFA reductions, including splenium of the CC. Females vs males: lower GFA in the CC, L SLF II and III, bilat frontostriatal and R IFOF
van Ewijk et al., 2014	170	17.3 (3.3) [8-30]	115	97.8 (14.7)	96	50	17	107	16.4 (3.1)	52	104.5 (13.7)	TBSS	Reduced FA in the L ATR and ILF; bilat CST, IC and SLF; R IFOF; CC (body, slenium, isthmus), forceps major; L temporal WM. Increased MD in the R CST, ILF, IFOF, SLF and IFOF. ASSOC: increased FA and reduced MD in widespread areas were associated with symptom severity
van Ewijk et al., 2015	1) whole ADHD sampe: 113	[14-24]	NA	NA	NA	NA	NA	73	[14-24]	NA	NA	TBSS	Widespread areas of reduced FA, similar to previous findings in a partly overlapping sample (van Ewijk et al., 2014)
van Ewijk et al., 2017	1) whole ADHD sample: 187 2) risk genotype (S/S): 40 3) other genotype (S/L): 147	[8–26]	1) 126 2) 26 3) 100	NA	NA	NA	NA	1) 103 2) 20 3) 83	[8–26]	1) 51 2) 9 3) 42	NA	TBSS	Reduced FA and MD in widespread WM areas similar to previous findings in a partly overlapping sample (van Ewijk et al., 2014)
Versace et al., 2021	126	34.3 (3.6)	113	100.1 (14.9)	NA	NA	NA	58	33.9 (4.1)	53	110.2 (13.2)	TG	Reduced FA in the L ILF and bilat CING. ADHD persisters had lower FA in the L ILF than remitters
Wang et al., 2021	42	32.12 (10.37)	23	NA	NA	NA	NA	59	31.85 (9.43)	32	NA	TG + network	Lower global efficiency and reduced density of rich-clubs among structural hub nodes, including the bilat precuneus, insula, caudate, L putamen, and R calcarine
Wolfers et al., 2015	101	35.83 (11.16)	38	108.08 (14.92)	NA	12	16	96	36.17 (11.17)	41	109.68 (15.34)	TBSS	ASSOC: FA in the R SLF and RTV
Wolfers et al., 2017	87	32.9 (9.5)	27	109.4 (15.9)	NA	NA	NA	93	35.1 (11.7)	27	107.8 (14.9)	TBSS + multimodal	Main multimodal marker of ADHD linked to morphological and microstructural effects within anterior temporal brain regions.
Yoncheva et al., 2016 (adults)	42	31.65 (9.8)	24	111.1 (11.8)	ADHD-C: 23 ADHD-I: 18	7	14	65	31.06 (9.0)	42	110.9 (10.6)	TBSS	Reduced global FA, AD, MD and MA. ASSOC: global MA and symptom severity.

Clinicodemographic characteristics, imaging analysis method, and main findings are reported. References are listed in Supplementary material.

*AD* axial diffusivity, *ADHD* Attention-deficit/hyperactivity disorder, *ADHD-C* ADHD combined presentation, *ADHD-HI* hyperactive/impulsive presentation, *ADHD-I* ADHD inattentive presentation, *ASSOC* association, *ATR* anterior thalamic radiation, *Bilat* bilateral, *CC* corpus callosum, *CING* cingulum, *CR* corona radiata, *CST* corticospinal tract, *EC* external capsule, *FA* fractional anisotropy, *FAT* frontal aslant tract, *FS* frontostriatal tract, *GFA* generalized fractional anisotropy, *HI* hyperactive/impulsive, *IC* internal capsule, *IFG* inferior frontal gyrus, *IFOF* inferior fronto-occipital fasciculus, *ILF* inferior longitudinal fasciculus, *IQ* intelligent quotient, *L* left, *MA* mode of anisotropy, *MCP* middle cerebellar peduncle, *MD* mean diffusivity, *MFG* middle frontal gyrus, *MOG* middle occipital gyrus, *MTG* middle temporal gyrus, *NA* not available, *OR* optic radiation, *PostCG* postcentral gyrus, *PreCG* precentral gyrus, *R* right, *RD* radial diffusivity, *ROI* region of interest, *RTV* reaction time variability, *SFG* superior frontal gyrus, *SLF* superior longitudinal fasciculus, *STG* superior temporal gyrus, *TBSS* tract-based spatial statistics, *TG* tractography, *TR* thalamic radiation, *UNC* uncinate, *VBA* voxel-based analysis, *WM* white matter.

#### Projection pathways

##### Fronto-striato-thalamic circuits

Most studies focused on fronto-striato-thalamic circuits (Fig. 2). Thirty-two studies reported tract metric alterations within distinct components of the frontostriatal pathways (connecting the frontal white matter to the striatum through the corona radiata and internal capsule) of children with ADHD as compared to controls [19–50]. Reduced FA was reported by seven tractography [23, 24, 27, 29, 36, 41, 45], four TBSS [25, 28, 37, 46], four VBA [19, 32, 40, 49] and two ROI [33, 38] studies. Increased FA was reported by only five studies, which either used a VBA [26, 35, 39] or a TBSS approach [43, 44]. Finally, four studies identified differences between ADHD presentations in children [34, 48, 51, 52]. Considering adult/mixed samples, 10 studies reported diffusion alterations in individuals with ADHD as compared to controls [53–62]. Of these, five reported reduced FA [55, 57, 58, 60, 62] and only one increased FA [53]. Further, differences in frontostriatal tracts were reported between ADHD persisters (i.e., those with a childhood diagnosis persisting in adulthood) and remitters [63]. Several studies also investigated brain-behavior relationships (Fig. 2).

Considering the thalamus and the anterior thalamic radiation (ATR), 13 studies reported significant case-control differences in children [20–22, 28, 30, 43, 50, 64–69]. Among these, two reported reduced FA [67, 68], and two increased FA [43, 66]. In adult/mixed samples, alterations were only identified by seven studies [56, 58, 70–74], of which four reported reduced FA [58, 70–72] and one both reduced and increased FA [74]. In summary, reduced FA was the most consistently reported alteration within fronto-striato-thalamic circuits, often observed bilaterally. Tract metrics were significantly correlated with both clinical and neuropsychological characteristics (Fig. 2).

##### Corticospinal tract (CST)

Fifteen studies reported diffusion alterations within the posterior limb of the internal capsule/CST in children with ADHD [25, 37, 38, 44, 48, 66, 67, 75–82]. Among these, reduced FA was noted by four TBSS [25, 37, 67, 75], one tractography [82] and one ROI study [80]. Increased FA was only identified by two TBSS [44, 66] and one tractography study [76]. Only two studies identified alterations in adult/mixed samples and either reported increased [74] or decreased FA in the CST [58]. In sum, the CST has been mainly investigated in children and most studies have reported reduced FA. Diffusion metrics have been associated with clinical symptoms but also task performance (Fig. 2).

##### Cerebellar pathways

Eight studies reported alterations in the cerebellum or the middle cerebellar peduncle (MCP) in children with ADHD [19, 20, 25, 32, 37, 49, 64, 83]. Among these, reduced FA was identified by three VBA [19, 32, 49], two TBSS [25, 37] and one ROI study [83]. No study reported increased FA. Only two studies identified alterations in the cerebellum/MCP of adults with ADHD [56, 74]. In sum, reduced FA was the most consistently reported alterations within the cerebellum/MCP. Only one study reported reduced FA in the inferior cerebellar peduncle [37]. Tract metrics were significantly associated with symptoms and cognitive performance (Fig. 2).

#### Commissural pathways

The most investigated commissural pathway was the CC (Fig. 3). We identified 17 studies that reported tract metric alterations in the CC of ADHD children as compared to controls [20, 21, 25, 28, 40, 43, 46, 48, 67, 75, 77, 84–89]. Reduced FA was found in seven TBSS studies [25, 28, 46, 67, 75, 84, 88] and in one VBA study [40], especially in the splenium of the CC. ROI studies reported conflicting results, with either increased [20] or decreased FA in the splenium of the CC [85]. Tractography studies reported reduced FA [86, 89]. Differences in diffusion metrics were also noted among ADHD presentations [48, 51] and treated/untreated individuals [87]. Eleven adult/mixed sample studies reported tract metric alterations in the CC [53–55, 57, 58, 70, 71, 74, 90–92]. Reduced FA was found in four TBSS studies especially in the body and splenium of the CC [55, 58, 70, 91], two VBA [54, 71] and an ROI study [92]. Finally, a tractography study in a mixed pediatric-adult sample reported reduced FA in the splenium of the CC [57], and another reduced FA in callosal fibers [60]. Overall, reduced FA was the most consistently reported alteration, especially in the splenium of the CC. Tract metrics were significantly correlated with both clinical and neuropsychological characteristics (Fig. 3).

#### Association pathways

##### Superior longitudinal fasciculus (SLF)

Seventeen studies reported tract metric alterations in the SLF of ADHD children as compared to controls [21, 23, 24, 37, 38, 46, 48, 50, 65, 70, 77, 78, 80, 84, 88, 93, 94]. Among these, reduced FA was observed in five TBSS [37, 46, 70, 84, 88], two tractography [23, 24], and one ROI study [80]. Only a TBSS study reported increased FA [94]. Differences in tract metrics were also observed between ADHD presentations [48, 51] and sexes [88]. Nine adult/mixed sample studies [53, 55, 58, 60, 71, 74, 95–97] reported case-control differences in SLF metrics. Of these, three TBSS [58, 70, 95], one tract-based analysis [97], one tractography [60], and one ROI study [96] noted reduced FA in individuals with ADHD as compared to controls. Further, a study combining an ROI and machine learning approach identified both reduced and increased FA in the SLF of adults with ADHD [74]. Sex differences were also observed by a tractography study in a mixed pediatric-adult sample, with lower FA in females [57]. Overall, reduced FA was the most consistently reported alteration, bilaterally or in either hemisphere with comparable frequency. Tract metrics were significantly correlated with both symptom severity and cognitive performance (Fig. 3).

##### Cingulum bundle

Twenty-one studies in children reported tract alterations in the cingulum of ADHD patients as compared to controls [21–24, 34, 37–39, 47, 48, 50, 66, 67, 69, 78, 88, 94, 98–101]. Reduced FA was noted by three TBSS [67, 88, 99] and four tractography studies [23, 24, 100, 101]. Increased FA was observed in two TBSS [66, 94] and two VBA studies [39, 98]. Differences were also identified among ADHD presentations [51, 52]. Eight studies in adult/mixed samples reported tract alterations in the cingulum [53, 54, 59, 71, 73, 74, 96, 102]. Reduced FA was noted by two VBA studies [54, 71], one tractography [102] and one ROI study [96]. Conversely, one VBA study reported increased FA [53] and one ROI study both increased and reduced FA [74]. Overall, both increased and reduced FA in the cingulum have been reported, bilaterally or in either hemisphere with equal frequency. Diffusion characteristics have been associated with variation in both clinical and cognitive profiles (Fig. 3).

##### Uncinate fasciculus

Ten studies in children and three in adults reported tract metric alterations in the uncinate fasciculus of individuals with ADHD as compared to controls [37, 38, 43, 53, 71, 74, 82, 87, 94, 97]. Of these, TBSS studies either reported increased [43, 94] or decreased FA [37, 82] in ADHD children. In adults, two VBA study reported increased FA [53, 71], whilst a tract-based analysis observed reduced FA [97]. Overall, main differences were observed either bilaterally or in the left uncinate, and were associated with inattentive-emotional symptoms, and cognitive deficits (Fig. 3).

##### Inferior longitudinal fasciculus (ILF)

Thirteen studies reported case-control differences in the ILF of ADHD children versus controls [21, 25, 37, 38, 46, 66, 75, 78, 79, 82, 87, 94, 103]. Reduced FA in the ILF was noted by four TBSS [25, 37, 46, 75], an ROI [103], and two tractography studies [79, 82]. Two studies reported increased FA [66, 94]. Differences were also identified between females and males [88] and between treated and untreated individuals [87]. Five studies in adult/mixed samples reported case-control differences in the ILF [58, 60, 74, 102, 104]. Using different techniques, three of these studies observed reduced FA [58, 102, 104], one increased FA [60], and one both increased and reduced FA [74]. Overall, reduced FA was the most frequently reported tract alteration, either bilaterally or in the left hemisphere, and was often associated with inattention (Fig. 3).

##### Inferior fronto-occipital fasciculus (IFOF)

Eleven studies in pediatric samples reported case-control differences in the IFOF [21, 43, 46, 48, 67, 75, 78, 79, 82, 84, 103]. Reduced FA was noted in four TBSS studies [46, 67, 75, 84], an ROI [103], and two tractography studies [79, 82]. Increased FA was only reported by one TBSS study [43]. Six studies reported case-control differences in the IFOF in adult or mixed children-adult samples [53, 58, 71, 74, 97, 104]. Of these, three studies using different techniques reported reduced FA [58, 74, 97] and one both reduced and increased FA [71]. Sex differences were also noted in a mixed pediatric-adult sample [57]. Overall, as for the ILF, reduced FA was the most frequently reported tract alteration, either bilaterally or in the left hemisphere, and was associated with inattention and emotional problems (Fig. 3).

### Quality assessment

Among the included studies, 68.7% were judged to be of low overall quality, mainly due to factors related to pre-processing (rated low-quality in 54% of studies) and/or acquisition parameters (rated low-quality in 46% of reports). Low-quality ratings for preprocessing were mainly due to lack of motion correction/quality checks; those for acquisition to the use of non-isotropic voxels or lack of information. The quality assessment of individual studies is reported in Supplementary Tables S4 and 5.

### Meta-analyses and meta-regressions

#### Meta-analysis of TBSS studies comparing ADHD versus controls

**Fig. 4:**
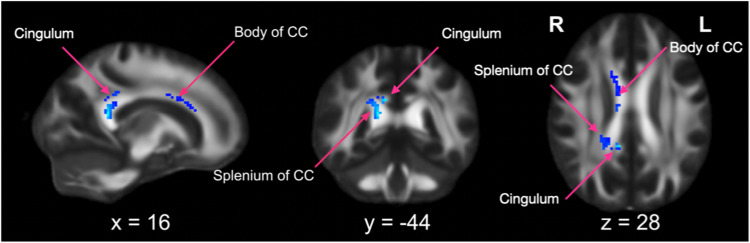
Results of the meta-analysis of TBSS studies. The meta-analysis of TBSS studies comparing fractional anisotropy (FA) between individuals with ADHD (any age) and typically developing (TD) controls showed that individuals with ADHD had lower FA in the splenium of the corpus callosum (CC), extending to the posterior cingulum, and in the body of the CC. Peak coordinates are reported in Supplementary Table S6.

Of the 43 TBSS studies included in the systematic review, 25 had available peak coordinates for case-control differences in FA and 18 in MD from non-duplicated datasets (see Supplementary Table S2). Therefore, these studies were included in two separate meta-analyses. The first meta-analysis included 25 TBSS studies, for a total of 32 datasets (26 in children and 6 in adults), comparing FA between 1348 individuals with ADHD and 1354 TD controls. As shown in Fig. 4 and Supplementary Table S6, the SDM analysis identified two clusters showing reduced FA values in the ADHD group compared with the TD control group. The right splenium of the CC, extending to the posterior cingulum, showed the most significant effect size and cluster extent (Fig. 4). The right body of CC exhibited the second-largest effect size and cluster extent. No clusters exhibited increased FA values in the ADHD group as compared with TD controls. The second meta-analysis included 18 studies, for a total of 23 datasets (19 in children and 4 in adults), comparing MD between 1051 participants with ADHD and 1101 controls, and did not identify any significant clusters. Due to the limited number of studies reporting peak coordinates for AD and RD (three and four, respectively), it was not possible to carry out meta-analyses for these metrics. Finally, the systematic review identified 16 studies that used a VBA approach. However, among these, only one study with available peak coordinates was not included in the previous review by Aoki et al., 2018 [11]. Therefore, we did not re-run the meta-analysis based on our pre-published protocol.

#### Meta-analyses of TBSS studies in children and adult samples

The meta-analyses of TBSS studies investigating case-control differences in FA in the pediatric sample (<18 years) showed no significant clusters. Similarly, we did not observe significant clusters when we separated children (<12 years) and adolescents. By contrast, the meta-analysis including only adult studies identified five clusters showing reduced FA in individuals with ADHD as compared to controls (Supplementary Table S7). The right splenium of the CC exhibited the largest effect size and cluster extent, and the right ATR showed the second-largest effect size and cluster extent. No clusters exhibited increased FA values in adults with ADHD as compared to controls. No significant cluster was identified when repeating the meta-analysis on case-control comparisons in MD separately in children and adults.

#### Meta-analysis of high-quality studies

In the sensitivity analysis considering only the 6 TBSS datasets rated of high quality (Supplementary Table S2), we did not observe significant differences in FA between ADHD and TD controls.

#### Meta-regression analyses

We ran three meta-regressions including the 25 TBSS studies (32 datasets) comparing FA between ADHD and TD controls. The first meta-regression tested the linear influence of age and identified two clusters with significant negative associations. In the right splenium and body of the CC, the differences between groups in reduced FA amplified with advancing age (Supplementary Table S8). No clusters displayed significant positive associations with age. These findings held after including treatment exposure as additional regressor (Supplementary Table S9). Meta-regressions accounting for the percentage of medication-naïve participants or the percentage of males included 23 studies (30 datasets) and 24 studies (31 datasets), respectively. We observed that ratios of medication-naïve/medicated individuals and males/females had no significant influence on FA.

## Discussion

We conducted the most comprehensive systematic review of DWI studies in the field of ADHD, meta-analyzing studies amenable to quantitative synthesis. The systematic review identified widespread alterations (mainly reduced FA) in individuals with ADHD as compared to controls, mostly in the fronto-striatal pathways, cingulum, and CC. The meta-analysis of TBBS studies included 32 datasets (of which six in adults) and identified the most consistent FA reduction in the right splenium (extending to the posterior cingulum), followed by the body, of the CC. Meta-regressions showed that these effects were not affected by sex or exposure to ADHD medication. However, lower FA was related to older age, and case-control differences did not survive in the pediatric meta-analysis. Conversely, the meta-analysis in adults mainly identified reduced FA in the right splenium of the CC and ATR.

Alterations in the splenium and the body of the CC are in line with their roles in supporting cognitive and motor functions affected in ADHD. The splenium of the CC connects the occipital, temporal and posterior parietal lobes of the two hemispheres [105] and has been associated with visuospatial information transfer, processing speed, IQ, and behavior [106]. Posterior cortical regions underpin attention and fronto-parietal cognitive control networks [107, 108], and thus contribute to cognitive functions, such as attention, working memory and executive control, which are commonly impaired in ADHD [109, 110]. Further, the body of the CC primarily connects premotor, supplementary and primary motor cortices between the two hemispheres, and contributes to the modulation of motor activity [105]. Our findings are in line with previous meta-analyses of structural magnetic resonance imaging (MRI) data reporting consistent volumetric reductions in the splenium of the CC in individuals with ADHD as compared to controls [111, 112]; although more recent meta- and mega-analyses observed significant case-control differences only in children [113, 114]. Further, functional MRI meta-analyses have identified reduced parietal and temporal activations during cognitive control, attention and timing tasks [115–118]. Of note, the cluster located in the splenium of the CC extended to the cingulum, which connects regions subserving the default mode network (DMN) [119]. There is fMRI evidence for a poor anticorrelation between the DMN and task-positive networks, such as the fronto-parietal control and ventral attentive networks [120, 121]. This, according to the default mode interference theory of ADHD [122], may cause lapses of attention during cognitively demanding tasks [123, 124]. Notably, a prior study reported that white matter disruption in the splenium and body of the CC was associated with decreased resting-state functional connectivity in the DMN posterior cingulate cortex [125]. Taken together, our findings and prior studies suggest that altered anatomical connectivity within the splenium and body of the CC may disrupt the function of brain networks supporting cognitive and motor functions affected in ADHD or their interaction with the DMN.

Importantly, our findings not only confirm but also extend those of previous meta-analyses of DWI studies. Although the first meta-analysis combining nine VBA and TBSS studies reported more consistent alterations within fronto-striato-cerebellar connections, subsequent meta-analyses mainly identified altered FA within commissural fibers [11, 12, 126]. The first included ten TBSS studies and reported reduced FA in the splenium of the CC, right sagittal stratum and left tapetum, extending to the cingulum, ILF and IFOF [12]. The second performed two separate meta-analyses, including 12 TBSS and 13 VBA studies respectively. It confirmed a consistent reduced FA in the CC, in addition to the ILF, IFOF and SLF. However, the meta-analysis of VBA studies also identified regions of increased FA, e.g., in the midcingulate and anterior CC [11]. Finally, a more recent meta-analysis combining 24 TBSS and VBA studies confirmed a consistent FA reduction in the splenium of the CC, extending to the body and right posterior corona radiata [126]. The high consistency among the more recent meta-analyses may be due to their largely overlapping pool of included studies (e.g., almost 90% of articles included in Zhao et al., 2022 [126] were included in Aoki et al., 2018 [11]). Our findings from 32 TBSS datasets further support the consistently reported callosal alterations in ADHD extending to the cingulum. Different mechanisms may lead to reduced FA, such as altered myelination, axonal density/diameter or fiber crossing [127, 128]. As FA is a composite measure, we encourage future studies to consistently report additional metrics, e.g. AD and RD, as this may help understand whether white matter alterations are primary (e.g. related to myelination) or secondary to those in the gray matter from which the tract origins from (e.g. number of neurons). Both these mechanisms have been suggested as plausible in relation to ADHD pathophysiology [113, 129]. Further, both genetic and environmental factors may potentially contribute to the observed alterations. For instance, a recent genome-wide association (GWA) meta-analysis in ADHD identified, among others, genes related to myelination [130]. However, their patter of methylation, which may reflect environmental adversity, affect ADHD symptoms trajectories [131]. Thus, genetic and environmental factors may interplay to cause white matter abnormalities, which in turn are associated with ADHD symptoms. Overall, there are likely multiple alternative pathophysiologic pathways underpinning ADHD and brain alterations are not necessarily causal to symptoms, but might be co-occurrent manifestations or consequence of ADHD behaviors.

As reported by Chen et al. [12], we also observed a negative association between FA and age, with lower FA values in older individuals. However, in this prior meta-analysis, findings in the splenium of the CC survived in the pediatric meta-analysis, in contrast to our study. The absence of significant findings in our meta-analysis restricted to children is somehow unexpected given that prior structural MRI meta- and mega-analyses reported significant case-control volumetric and morphometric differences in children but not in adults with ADHD [113, 114]. For instance, reduced surface area and cortical thickness were identified in fronto-cingulate-temporo-occipital regions of ADHD children, but not in adults, compared to controls [114]. However, we believe that our findings could provide new important insight into the pathophysiology of ADHD across the lifespan. A possible explanation of differences between prior and our results might be related to the distinct developmental trajectories of the gray and white matter. While gray matter structural measures increase and reach their peak in childhood (~age 2 for cortical thickness; ~age 6 for gray matter volume; and ~11-12 years for surface area/cerebral volume) and then decrease in a curvilinear fashion [132]; FA in the CC increases in a curvilinear fashion with age, peaking between 21 and 29 years [133, 134]. Hence a delayed, less steep increase in FA with age in ADHD compared to healthy subjects would be reflected in larger case-control differences in older subjects; whilst a less steep decrease in gray matter measures would be more pronounced in childhood. The reduction in FA we observed in the splenium and body of the CC of individuals with ADHD could hence reflect a delay in white matter maturation, parallel to that observed in posterior cortices [135], which are interhemispherically connected by the posterior CC. However, longitudinal studies investigating developmental trajectories are needed to clarify the course of brain structure and connectivity alterations in ADHD.

We also observed a lack of spatial convergence in the sensitivity analysis only including high-quality studies. These findings need to be interpreted with caution because, although the quality assessment was based on published recommendations, there is not an available, established gold standard quality rating tool for DWI studies. Further, this meta-analysis only included six studies. Nevertheless, these findings suggest that the case-control differences identified in DWI meta-analyses might be influenced by non-optimal acquisition parameters or preprocessing. Future studies should be encouraged to follow the existing recommendations on imaging data acquisition, preprocessing, and analysis (referenced in Supplementary Table S3). For example, studies should (1) opt for isotropic voxels to avoid bias; (2) include sufficient gradient directions to ensure rotation invariance and improve precision; (3) spread multiple gradients unweighted among weighted volumes in the scan for similar noise profiles; (4) report sufficient acquisition and preprocessing information; (5) include additional scans for distortion correction; and (6) perform data quality control on the motion and report motion thresholds. Further, research in ADHD may benefit from methodological advances in the field. For instance, higher encoding resolution may improve sensitivity to signal and biophysical properties [136]; acceleration techniques may improve applications to younger populations [137]; and newer preprocessing methods may handle more types of artefacts [138]. Overall, methodological developments may improve the study quality and reliability of findings, thus fostering our understanding of brain microstructure and connectivity in ADHD.

Prior meta-analyses of ADHD studies in other imaging modalities also reported no significant spatial convergence [139, 140], and related this to methodological differences among studies and the high clinical and neurobiological heterogeneity of ADHD. In support to the latter observation, our systematic review indicates that differences may exist between ADHD presentations, females and males, treated versus untreated individuals, and participants with/without comorbidities. Further, a recent meta-analysis has shown that FA reduction in the splenium of the CC is a common feature of both ADHD and ASD, although the latter is characterized by additional white matter alterations in frontostriatal pathways [126]. These findings, together with the limited converging results in the whole sample meta-analysis and the absence of significant results in the pediatric meta-analysis, should encourage future studies to extend their investigations beyond case-control differences and determine whether subgroups of individuals with ADHD could be discerned based on white matter characteristics. The need to parse neuroanatomic heterogeneity has also been raised by studies in other MRI modalities [141], as this may improve our understanding of ADHD pathophysiology. Further, the stratification of the heterogenous ADHD population based on differences in the underlying neuroanatomy may pave the way to the development of new more targeted treatments (Parlatini et al., under review).

### Limitations

Some limitations of this work should be considered, mostly related to limitations of the included studies. For instance, most studies included in the narrative review used traditional tensor-based methods, and only a minority included other imaging (e.g., q-ball) and analysis (e.g., graph theory) techniques. We encourage the use of state-of-the-art methods as this can also advance our understanding of ADHD. As in previous meta-analyses, we used a coordinate-based approach rather than the original t-statistic maps, as these are not publicly available, but this may limit the accuracy of the results [14]. We conducted meta-analyses of TBSS studies but did not replicate the one of VBA studies, according to our pre-published protocol, as we could only have included one additional study compared to Aoki et al. [11]. This reflects the general tendency in the current literature towards the use of TBSS as compared to VBA approaches. As in this previous work [11], we preferred not to combine TBSS and VBA approaches in a single meta-analysis because this would violate the assumption under the null hypothesis that the expected FA differences are equal at every voxel, and because there is evidence that the two approaches may produce non-converging findings [11]. We conducted separate meta-analyses of FA and MD, but could not investigate other diffusion metrics (e.g., RD and AD) due to the limited number of studies reporting them. We contacted authors for missing data, but peak coordinates were not available for eight studies. Our meta-analysis included a much greater number of studies than prior syntheses, which enhances robustness of findings; however, we encourage future investigations to provide full details of their results to limit potential reporting bias.

The studies included in the systematic review/meta-analyses mostly recruited small samples and were heterogeneous in terms of clinicodemographic characteristics. They included varying proportions of males and females and different ADHD presentations. Most studies included subjects previously exposed to medication, which can represent a potential confounder in connectivity measurements [16]. Our meta-regression analysis did not show any significant effect of previous exposure to stimulants; however, longitudinal studies in large samples are needed to disentangle the effects of development and treatment. Further, only few studies restricted the recruitment to comorbidity-free individuals. Most included different disorders or did not provide information, thus we were unable to control for comorbidities. This heterogeneity may have potentially influenced consistency among studies and our findings. For instance, about one fifth of individuals with ADHD also have ASD [142]. However, comorbidity was not officially allowed until the DSM-5 was published (2013) [1]; therefore, the most recent studies and those in pediatric samples may be enriched with comorbid cases. This may also have potentially contributed to the lack of convergent findings in our pediatric submeta-analysis. For instance, recent meta-analyses highlighted both shared and specific alterations in individuals with ADHD and ASD [126, 143]. One of these identified reduced FA in the CC of both ADHD and ASD individuals, but also clusters of increased FA in those with ASD [126]. This may have confounded and could be responsible for the negative findings in children with ADHD. Finally, comorbidities, such as affective and substance use disorders, are more common in adults with ADHD [5] and could also have confounded the results. Restricting recruitment to comorbidity-free individuals does not reflect daily clinical practice and limits our understanding of the biological basis of brain disorders. An alternative for future studies is that of taking a dimensional approach, in line with the National Institute of Mental Health Research Domain Criteria (RDoC) framework [5, 144]. In our meta-analysis, we considered the potential effect of sex and treatment exposure on the findings. However, the number of studies comparing ADHD presentations was limited, thus we could not meta-analyze them, and this should be subject of future investigations. Finally, we separately analyzed studies in children and adults, which allowed us to identify adult-specific alterations; however, the number of studies in the adult population was limited, thus findings should be interpreted with caution. Nevertheless, the association we observed between callosal FA and age, as well as findings from previous structural MRI studies, should encourage the longitudinal investigation of differences in the developmental trajectories of the white and gray matter in ADHD.

## Conclusions

Clinicodemographic and methodological differences among studies are major barriers to our understanding of the neurobiology of ADHD. Future studies are needed to disentangle the potential biological differences related to sex, age, presentations, and comorbidities. They may also investigate tracts that have so far received less attention, such as the cerebellar peduncles, especially in adults. Finally, methodological improvements are recommended, especially optimizing imaging parameters, controlling and reporting for head motion, as well as efforts to enhance the comparability among studies, e.g., by using anatomical atlases to identify ROIs and tracts. While currently DWI studies do not have direct clinical implications in the field of ADHD [145], our findings may help future studies to stratify individuals with ADHD according to the underlying pathophysiology, which may guide the development of more tailored treatments. To this purpose, future study may also benefit from the combination of multiple imaging approaches, e.g., fMRI, functional Near Infrared Spectroscopy (fNIRS), and positron emission tomography (PET), as these can provide complementary information on functional and metabolic changes associated with ADHD.

### Supplementary information

Supplementary material

PRISMA checklist

Peak coordinates -1

Peak coordinates -2
