# Supplementary material for: White matter alterations in Attention-Deficit/Hyperactivity Disorder (ADHD): a systematic review of 129 diffusion imaging studies with meta-analysis
Source: Mol Psychiatry. 2023 Jul 21;28(10):4098–123. doi: 10.1038/s41380-023-02173-1 (PMC10827669; doi:10.1038/s41380-023-02173-1)
Supplement: Supplementary file 1 — Supplementary material [file 41380_2023_2173_MOESM1_ESM.docx]

**SUPPLEMENTARY MATERIAL**

**TABLE OF CONTENT**

**Syntax and results from each database page 2**

**Reports excluded from the systematic review and meta-analysis page 3**

Table S1. TBSS studies excluded from the meta-analysis page 3

**TBSS studies included in the meta-analyses and meta-regressions page 4**

Table S2. TBSS studies included in meta-analyses and meta-regressions page 4

**Associations between diffusion metrics and ADHD symptoms/cognitive deficits** **page 5**

**Quality assessment page 7**

Table S3. Criteria to assess imaging data quality page 7

Table S4. Quality assessment of the imaging studies in children page 9

Table S5. Quality assessment of the imaging studies in adult/mixed samples page 15

**Meta-analyses and meta-regressions page 18**

Table S6. Results of the meta-analysis of TBSS studies (FA) page 18

Table S7. Results of the meta-analysis of TBSS studies in adults (FA) page 18

Table S8. Results of the meta-regression: association between age and FA page 18

Table S9. Results of the meta-regression: testing age and treatment exposure effects page 19

**REFERENCES page 20**

**Studies included in the systematic review page 20**

**Studies included in the supplementary material page 30**

***Syntax and results from each database***

PUBMED (MEDLINE)

(ADHD [tiab] OR adhd [tiab] OR attention deficit disorder with hyperactivity [tiab] OR syndrome hyperkinetic [tiab] OR hyperkinetic syndrome [tiab] OR hyperactivity disorder [tiab] OR hyperactive child syndrome [tiab] OR childhood hyperkinetic syndrome [tiab] OR attention deficit hyperactivity disorders [tiab] OR attention deficit hyperactivity disorder [tiab] OR attention deficit hyperactivity disorder [tiab] OR adhd [tiab] OR overactive child syndrome [tiab] OR attention deficit hyperkinetic disorder [tiab] OR hyperkinetic disorder [tiab] OR attention deficit disorder hyperactivity [tiab] OR child attention deficit disorder [tiab] OR hyperkinetic syndromes [tiab] OR syndromes hyperkinetic [tiab] OR hyperkinetic syndrome childhood [tiab]) AND (tensor [tiab] OR tract-based spatial statistics [tiab] OR TBSS [tiab] OR DTI [tiab] OR fractional anisotropy [tiab] OR tractography [tiab] OR diffusion [tiab] OR spherical deconvolution [tiab])

Limits: none

Results: 276

OVID databases (PsycInfo, EMBASE+EMBASE classic, OVID Medline)

(ADHD OR adhd OR attention deficit disorder with hyperactivity OR syndrome hyperkinetic OR hyperkinetic syndrome OR hyperactivity disorder OR hyperactive child syndrome OR childhood hyperkinetic syndrome OR attention deficit hyperactivity disorders OR attention deficit hyperactivity disorder OR adhd OR overactive child syndrome OR attention deficit hyperkinetic disorder OR hyperkinetic disorder OR attention deficit disorder hyperactivity OR child attention deficit disorder OR hyperkinetic syndromes OR syndromes hyperkinetic OR hyperkinetic syndrome childhood) AND (tensor OR tract-based spatial statistics OR TBSS OR DTI OR fractional anisotropy OR tractography OR diffusion OR spherical deconvolution) [tiab].

Limits: none

Results: 1553

WEB OF KNOWLEDGE (Web of science (science citation index expanded), Biological abstracts, Biosis, Food science and technology abstracts)

(ADHD OR adhd OR attention deficit disorder with hyperactivity OR syndrome hyperkinetic OR hyperkinetic syndrome OR hyperactivity disorder OR hyperactive child syndrome OR childhood hyperkinetic syndrome OR attention deficit hyperactivity disorders OR attention deficit hyperactivity disorder OR adhd OR overactive child syndrome OR attention deficit hyperkinetic disorder OR hyperkinetic disorder OR attention deficit disorder hyperactivity OR child attention deficit disorder OR hyperkinetic syndromes OR syndromes hyperkinetic OR hyperkinetic syndrome childhood) AND (tensor OR tract-based spatial statistics OR TBSS OR DTI OR fractional anisotropy OR tractography OR diffusion OR spherical deconvolution) (in Topic)

Limits: none

Results: 752

TOTAL=2581

After importing all in endnote (with “duplicate remove” function): 1387

After further manual de-duplication: 956

***Reports excluded from the systematic review and meta-analysis***

Following full-text screening, we excluded 34 reports from the systematic review.

Among these, 17 did not collect (separate) diffusion imaging data for both ADHD and controls (1-17). Further, 14 were not peer-reviewed original articles (18-31). Finally, three did not use diffusion imaging (32-34). Considering TBSS studies, 18 were excluded from the meta-analysis, as listed in Table S1 with reasons.

**Table S1. TBSS studies excluded from the meta-analysis.** Of note, authors of studies without available coordinates have been contacted but we did not receive reply/requested information from those listed below.

| **TBSS studies excluded from the meta-analysis (N=18)** | **Reasons** |
| --- | --- |
| Ameis et al., 2016 (35) | No coordinate table |
| Ball et al., 2019 (36) | No group comparison data |
| Basay et al., 2016 (37) | No coordinate table |
| Dramsdahl et al., 2012 (38) | No whole brain |
| Francx et al., 2015 (39) | No whole brain |
| Francx et al., 2016 (40) | No coordinate table |
| O'Neill et al., 2019 (41) | No coordinate table |
| Park et al., 2016 (42) | No coordinate table |
| Pastura et al., 2016 (43) | No coordinate table |
| Saad et al., 2021 (44) | No coordinate table |
| Tamm et al., 2012 (45) | No whole brain |
| van Ewijk et al., 2015 (46) | Duplicated sample |
| van Ewijk et al., 2017 (47) | Duplicated sample |
| Wolfers et al., 2015 (48) | No group comparison data |
| Wolfers et al., 2017 (49) | No group comparison data |
| Wu et al., 2019 (50) | Duplicated sample |
| Wu et al., 2020 (51) | Duplicated sample |
| Yoo et al., 2020 (52) | No coordinate table |

***TBSS studies included in the meta-analyses and meta-regressions***

**Table S2. TBSS studies included in meta-analyses and meta-regressions.** This table shows the number of available datasets reporting FA and MD in individuals with ADHD and controls of any age; FA in pediatric and adult samples, and FA in high-quality studies.

| **TBSS studies included in**  **meta-analysis** | **FA datasets** | **MD**  **datasets** | **Children datasets**** | **Adult**  **datasets** | **High quality datasets** |
| --- | --- | --- | --- | --- | --- |
| Adisetiyo et al., 2014 (53) | 1 | 1 | 1 (ad) | 0 | 0 |
| Aoki et al., 2017 (54) | 1 | 1 | 1 (ch) | 0 | 1 |
| Bessette et al., 2019 (55) | 1 | 0 | 1 (ad) | 0 | 0 |
| Bode et al., 2015 (56) | 1 | 1 | 0 | 1 | 0 |
| Bos et al., 2017 (57) | 1 | 1 | 1 (ch) | 0 | 0 |
| Bouziane et al., 2018 (58) | 2 | 0 | 1 (ch) | 1 | 0 |
| Chuang et al., 2013 (59) | 1 | 0 | 1 (ad) | 0 | 0 |
| Cooper et al., 2014 (60) | 1 | 1 | 1 (ad) | 0 | 0 |
| Cortese et al., 2013 (61) | 1 | 0 | 0 | 1 | 0 |
| de Luis-Garcia et al., 2015 (62) | 2 | 2 | 2 (ch) | 0 | 0 |
| Ercan et al., 2016 (63) | 2 | 0 | 2 (ch) | 0 | 0 |
| King et al., 2015 (64) | 1 | 0 | 1 (ad) | 0 | 1 |
| Nagel et al., 2011 (65) | 1 | 1 | 1 (ch) | 0 | 0 |
| O'Conaill et al., 2015 (66) | 1 | 1 | 1 (ch) | 0 | 0 |
| Ohta et al., 2020 (67) | 1 | 1 | 0 | 1 | 1 |
| Onnink et al., 2015 (68) | 1 | 1 | 0 | 1 | 0 |
| Rossi et al., 2015 (69) | 1 | 0 | 1 (ch) | 0 | 0 |
| Saenz et al., 2020 (70) | 1 | 1 | 1 (ch) | 0 | 0 |
| Silk et al., 2009 (71) | 1 | 1 | 1 (ad) | 0 | 0 |
| Svatkova et al., 2016 (72) | 2 | 2 | 2 (ad) | 0 | 0 |
| Unsel-Bolat et al., 2020 (73) | 2 | 2 | 2 (ch) | 0 | 0 |
| van Ewijk et al., 2014* (74) | 1 | 1 | 1 (ad) | 0 | 1 |
| Wu et al., 2017 (75) | 1 | 1 | 1 (ch) | 0 | 0 |
| Wu et al., 2022 (76) | 2 | 2 | 2 (ch) | 0 | 0 |
| Yoncheva et al., 2016 (77) | 2 | 2 | 1 (ch) | 1 | 2 |
| TOTAL N | 32 | 23 | 26 | 6 | 6 |

*Van Ewijk et al., 2014 included a mixed child-adult sample. As the mean age was 17 years, this study was included among those in pediatric samples.

** We divided the pediatric sample in children (<12 years) and adolescents (>12 and <18 years) based on the mean age reported in the studies (Table 1). Ad=adolescents, Ch=children.

**Associations between diffusion metrics and ADHD symptoms/cognitive deficits**

Fronto-striato-thalamic circuits

Most studies focused on fronto-striato-thalamic circuits (Fig. 2). In children, significant associations were noted with attention (78-82); impulsivity (55, 75, 79, 80, 82), executive functions and school problems (59, 83-86); symptom severity (83, 86-91) and improvement under treatment (92). In adults, associations were reported with symptom severity (93-96) and impulsivity (68). Considering the thalamus and the anterior thalamic radiation (ATR), associations were found with symptom severity (50, 91, 97), attention (78), and treatment response (92) in children; and with attention (98), impulsivity (99), and symptom severity (95) in adults.

Corticospinal tract (CST)

In children, significant associations were identified between CST metrics and ADHD symptom severity (50, 100), age-related improvement of hyperactive/impulsive symptoms (39), autistic traits (60, 101), adaptive functioning (35), impulsivity (55) and attention (62, 69, 100, 102). Only two studies identified alterations in adult/mixed samples and either reported increased (103) or decreased FA in the CST (74), without significant associations.

Cerebellar pathways

In children, significant associations were reported between cerebellar metrics and attention and cognitive performance (59, 104). Only two studies identified alterations in the cerebellum/middle cerebellar peduncle (MCP) of adults with ADHD (95, 103). Of this one reported a significant association between cerebellar network organization and symptom severity (95).

Corpus callosum (CC)

Considering anatomo-clinical correlations in children, diffusion metrics were shown to be associated with symptom severity (36, 54, 75, 100, 105), age-related improvement of hyperactive/impulsive symptoms (39), emotion dysregulation (105), adaptive functioning (35), delay aversion (55), working memory and motor control (100, 106). In adult/mixed samples, significant associations were reported between callosal metrics and sensory sensitivity (67), delay discounting (68), and cognitive performance (107).

Superior longitudinal fasciculus (SLF)

In children, significant associations were reported between SLF tract metrics and symptom severity (36, 50, 75, 82), age-related improvement of hyperactive/impulsive symptoms (39), emotion dysregulation (105), memory and executive functions (70, 83, 100), and motor control (106, 108). Similarly, in adult/mixed samples, SLF metrics were significantly associated with symptom severity (109, 110), attention (48, 99), and cognitive performance (107).

Cingulum bundle

In children, significant associations were identified between tract metrics and ADHD symptom severity (82, 89, 91, 101, 111), age-related remission of hyperactive-impulsive symptoms (39), treatment response (92), emotional problems (105, 112), working memory (70), attention (69, 79, 81, 82, 104), and executive functions (72, 113). In adult/mixed samples, significant associations were reported with symptom severity (93) and executive functions (107).

Uncinate fasciculus (UF)

In children, significant associations were reported between tract metrics and callous-unemotional behaviors (114), emotion dysregulation (105) and inattention (69); in adult/mixed samples significant associations were observed with inattentive symptoms (109), impulsivity (99); working memory, attention, and verbal intelligence (107).

Inferior longitudinal fasciculus (ILF)

In children, significant anatomo-clinical correlations were reported between tract metrics and symptom severity and executive functions (72), adaptive functioning (35), and emotion dysregulation (105). Similarly, in adult or mixed samples, significant associations were reported between tract metrics and inattentive symptoms (109) and attention performance (115).

Inferior fronto-occipital fasciculus (IFOF)

In children, significant correlations were reported between IFOF metrics and symptom severity (50), callous-unemotional behaviors (114), emotion dysregulation (105), adaptive functioning (35), and attention (69). Further, significant associations were observed with inattentive symptoms in adults (109).

***Quality assessment***

**Table S3. Criteria to assess imaging data quality.** These criteria were derived from published recommendations on acquisition, pre-processing and analysis.

|  | CRITERIA | Green | Amber | Red | References |
| --- | --- | --- | --- | --- | --- |
| Acquisition | Number of unique directions | > =60 | 30-59 | <30 /missing data | (116-119) |
|  | B-value | >=1000 | 700-1000 | <700/missing data | (119, 120) |
|  | Voxel size | Isotropic voxel |  | Non-isotropic voxel | (119) |
| Preprocessing | Exclusion due to motion | Motion check with exclusion criteria  (Visual check, group comparison, motion as covariate in regression, exclusion criteria, or automatic in-scanner check) | Mentioned motion correction with unclear effect  (Repeat scans with motion, but no clear effect) | No motion / data quality check  (No mention about motion) | (119, 121) |
|  | Eddy current correction and motion correction | Present  FSL with nonlinear correction, or  FSL eddy current, SPM8 (affine transformation + mutual information cost) with b<1500 |  | Missing or FSL eddy current, SPM8 (affine transformation + mutual information cost) with b>1500 | (119, 120, 122, 123) |
| Analysis | Voxel based versus manual ROI | VBA/TBSS |  | Manual delineation of ROI | (120, 124) |
|  | Correcting for multiple comparisons (if applicable) | Yes | Some steps only | Not at all | (125) |
|  | Resampling | Resampling before tractography for non-isotropic acquisition | No resampling before tractography when acquisition with non-isotropic voxel but no slice gap | No resampling before tractography when acquisition with slice gap and non-isotropic voxel | (119, 126, 127) |
|  | Normalization | Normalization for streamline count |  | No normalization in deterministic tracking and using streamline count | (119, 127) |
|  | Edge | Using microstructure or streamline count |  | Fibre count | (119, 127) |

**Table S4. Quality assessment of the imaging studies in children.**

**Abbreviations**

ANT=Advanced Normalization Tools**,** CATNAP=Coregistration, Adjustment, and Tensor-solving, a Nicely Automated Program**,** DIVE=diffusion imaging visualization environment**,** DKI=diffusion kurtosis imaging**,** DTI=diffusion tensor imaging, DSI=diffusion spectrum imaging, FACT=fractional anisotropy continuous tracking, FDT= FMRIB Diffusion Toolbox, FLIRT= FMRIB Linear Image Registration Tool, FMRIB=Functional Magnetic Resonance Imaging of the Brain, FSL= FMRIB software library, GQI=Generalized q-sampling imaging, HARDI=High Angular Resolution Diffusion Imaging, NA=not available, NS=not significant, PANDA=Pipeline for Analyzing braiN Diffusion imAges, PATCH=Patching ArTefacts from Cardiac and Head motion, PRIDE=Philips Research Imaging Development Environment, ROI= region of interest, SPM=statistical parametric mapping, TBSS= tract-based spatial statistics, TG=tractography, TRACULA=TRActs Constrained by UnderLying Anatomy, VBA=voxel-based analysis.

**Table S5. Quality assessment of the imaging studies in adult/mixed samples.**

**Abbreviations**

DTI=diffusion tensor imaging, DRAMMS=Deformable Registration via Attribute Matching and Mutual-Saliency , DSI=diffusion spectrum imaging, FLIRT= FMRIB Linear Image Registration Tool, FMRIB=Functional Magnetic Resonance Imaging of the Brain, FSL= FMRIB software library, NA=not available, NS=not significant, PANDA=Pipeline for Analyzing braiN Diffusion imAges, PATCH=Patching ArTefacts from Cardiac and Head motion, ROI= region of interest, SPM=statistical parametric mapping, TBSS= tract-based spatial statistics, TFCW=Threshold-free cluster weighted, TG=tractography, VBA=voxel-based analysis.

**META-ANALYSES AND META-REGRESSIONS**

| **Table S6.** **Results of the meta-analysis of TBSS studies (FA)** | | | | |
| --- | --- | --- | --- | --- |
| ADHD > TDC | | | | |
| MNI coordinates | *Z*-value | *p*-value | No. of voxels | Description |
| none | | | | |
| TDC > ADHD | | | | |
| 16, -44, 18 | -4.059 | < 0.0001 | 202 | Splenium of corpus callosum |
| 12, -14, 28 | -3.000 | 0.0014 | 64 | Body of corpus callosum |
| **Abbreviations:** ADHD: attention-deficit/hyperactivity disorder, FA: fractional anisotropy, MNI: Montreal Neurological Institute, TBSS: tract-based spatial statistics, and TDC: typically developing control. | | | | |
|  |  |  |  |  |
| **Table S7. Results of the meta-analysis of TBSS studies in adults (FA)** | | | | |
| ADHD > TDC | | | | |
| MNI coordinates | *Z*-value | *p*-value | No. of voxels | Description |
| none | | | | |
| TDC > ADHD | | | | |
| 22, -48, 24 | -3.534 | 0.0002 | 94 | Splenium of corpus callosum |
| 4, -18, 18 | -3.289 | 0.0005 | 20 | Anterior thalamic radiation |
| 14, -8, 32 | -2.910 | 0.0018 | 15 | Body of corpus callosum |
| 18, 20, 28 | -3.204 | 0.0007 | 14 | Anterior corona radiata |
| 12, -44, 28 | -3.048 | 0.0012 | 13 | Cingulum |
| **Abbreviations:** ADHD: attention-deficit/hyperactivity disorder, FA: fractional anisotropy, MNI: Montreal Neurological Institute, TBSS: tract-based spatial statistics, and TDC: typically developing control. | | | | |
|  |  |  |  |  |
| **Table S8. Results of the meta-regression: association between age and FA** | | | | |
| MNI coordinates | *Z*-value | *p*-value | No. of voxels | Description |
| Age-related increases in reduced FA | | | | |
| 20, -40, 30 | -3.758 | < 0.0001 | 127 | Splenium of corpus callosum |
| 12, 14, 24 | -3.588 | 0.0001 | 68 | Body of corpus callosum |
| **Abbreviations:** FA: fractional anisotropy; MNI: Montreal Neurological Institute | | | | |

**Table S9. Results of the meta-regression: testing age and treatment exposure effects**

We ran an additional analysis to test the potential confounding effect of treatment exposure in the meta-regression of age. As shown in the table, the results of the meta-regression of age held after adding treatment exposure as an additional regressor. This finding suggests that, according to our meta-regression, the potential confounding effect of treatment exposure was negligible across the age range.

| MNI coordinates | *Z*-value | *p*-value | No. of voxels | Description |
| --- | --- | --- | --- | --- |
| Age-related increases in reduced FA | | | | |
| 20, -40, 30 | -4.57 | < 0.0001 | 78 | Splenium of corpus callosum |
| 12, 12, 25 | -3.63 | 0.0001 | 149 | Body of corpus callosum |
| **Abbreviations:** FA: fractional anisotropy; MNI: Montreal Neurological Institute | | | | |

**REFERENCES OF STUDIES INCLUDED IN THE SYSTEMATIC REVIEW**

We included 96 studies in children (35-37, 39-45, 50-55, 57, 59, 60, 62-66, 69-73, 75, 76, 78-84, 86-92, 97, 100-102, 104-106, 108, 111-114, 128-166), and 32 in adult/mixed samples (38, 46-49, 56, 58, 61, 67, 68, 74, 77, 93-96, 98, 99, 103, 107, 109, 110, 115, 167-176).

1. Acer N, Dolu N, Zararsiz G, Dogan MS, Gumus K, Ozmen S, et al. Anatomical characterization of ADHD using an atlas-based analysis: A diffusion tensor imaging study. EuroBiotech J. 2017;1(1):46-56.

2. Adisetiyo V, Tabesh A, Di Martino A, Falangola MF, Castellanos FX, Jensen JH, et al. Attention-deficit/hyperactivity disorder without comorbidity is associated with distinct atypical patterns of cerebral microstructural development. [References]. Human Brain Mapping. 2014;35(5):2148-62.

3. Alger JR, O'Neill J, O'Connor MJ, Kalender G, Ly R, Ng A, et al. Neuroimaging of Supraventricular Frontal White Matter in Children with Familial Attention-Deficit Hyperactivity Disorder and Attention-Deficit Hyperactivity Disorder Due to Prenatal Alcohol Exposure. Neurotox Res. 2021.

4. Ameis SH, Lerch JP, Taylor MJ, Lee W, Viviano JD, Pipitone J, et al. A diffusion tensor imaging study in children with ADHD, autism spectrum disorder, OCD, and matched controls: Distinct and non-distinct white matter disruption and dimensional brain-behavior relationships. [References]. The American Journal of Psychiatry. 2016;173(12):1213-22.

5. Aoki Y, Yoncheva YN, Chen B, Nath T, Sharp D, Lazar M, et al. Association of white matter structure with autism spectrum disorder and attention-deficit/hyperactivity disorder. [References]. JAMA Psychiatry. 2017;74(11):1120-8.

6. Ashtari M, Kumra S, Bhaskar SL, Clarke T, Thaden E, Cervellione KL, et al. Attention-deficit/hyperactivity disorder: a preliminary diffusion tensor imaging study. Biological Psychiatry. 2005;57(5):448-55.

7. Ball G, Malpas CB, Genc S, Efron D, Sciberras E, Anderson V, et al. Multimodal Structural Neuroimaging Markers of Brain Development and ADHD Symptoms. American Journal of Psychiatry. 2019;176(1):57-66.

8. Basay BK, Buber A, Basay O, Alacam H, Ozturk O, Suren S, et al. White matter alterations related to attention-deficit hyperactivity disorder and COMT val158met polymorphism: Children with valine homozygote attention-deficit hyperactivity disorder have altered white matter connectivity in the right cingulum (cingulate gyrus). [References]. Neuropsychiatric Disease and Treatment. 2016;12:969-81.

9. Beare R, Adamson C, Bellgrove MA, Vilgis V, Vance A, Seal ML, et al. Altered structural connectivity in ADHD: a network based analysis. Brain Imaging Behav. 2017;11(3):846-58.

10. Bechtel N, Kobel M, Penner I-K, Klarhofer M, Scheffler K, Opwis K, et al. Decreased fractional anisotropy in the middle cerebellar peduncle in children with epilepsy and/or attention deficit/hyperactivity disorder: A preliminary study. [References]. Epilepsy & Behavior. 2009;15(3):294-8.

11. Bessette KL, Stevens MC. Neurocognitive Pathways in Attention-Deficit/Hyperactivity Disorder and White Matter Microstructure. Biol Psychiatry Cogn Neurosci Neuroimaging. 2019;4(3):233-42.

12. Bos DJ, Oranje B, Achterberg M, Vlaskamp C, Ambrosino S, de Reus MA, et al. Structural and functional connectivity in children and adolescents with and without attention deficit/hyperactivity disorder. J Child Psychol Psychiatry. 2017;58(7):810-8.

13. Bu X, Yang C, Liang K, Lin Q, Lu L, Zhang L, et al. Quantitative tractography reveals changes in the corticospinal tract in drug-naïve children with attention-deficit/hyperactivity disorder. J Psychiatry Neurosci. 2020;45(2):134-41.

14. Cao Q, Shu N, An L, Wang P, Sun L, Xia M-R, et al. Probabilistic diffusion tractography and graph theory analysis reveal abnormal white matter structural connectivity networks in drug-naive boys with attention deficit/hyperactivity disorder. [References]. The Journal of Neuroscience. 2013;33(26):10676-87.

15. Cao Q, Sun L, Gong G, Lv Y, Cao X, Shuai L, et al. The macrostructural and microstructural abnormalities of corpus callosum in children with attention deficit/hyperactivity disorder: A combined morphometric and diffusion tensor MRI study. [References]. Brain Research. 2010;1310:172-80.

16. Çelik Z, Çolak Ç, Di Biase MA, Zalesky A, Zorlu N, Bora E, et al. Structural connectivity in adolescent synthetic cannabinoid users with and without ADHD. Brain Imaging Behav. 2020;14(2):505-14.

17. Cha J, Fekete T, Siciliano F, Biezonski D, Greenhill L, Pliszka SR, et al. Neural Correlates of Aggression in Medication-Naive Children with ADHD: Multivariate Analysis of Morphometry and Tractography. Neuropsychopharmacology. 2015;03.

18. Chen L, Huang X, Lei D, He N, Hu X, Chen Y, et al. Microstructural abnormalities of the brain white matter in attention-deficit/hyperactivity disorder. [References]. Journal of Psychiatry & Neuroscience. 2015;40(4):280-7.

19. Chiang H-L, Chen Y-J, Lo Y-C, Tseng W-YI, Gau SS-F. Altered white matter tract property related to impaired focused attention, sustained attention, cognitive impulsivity and vigilance in attention-deficit/ hyperactivity disorder. [References]. Journal of Psychiatry & Neuroscience. 2015;40(5):325-35.

20. Chiang HL, Chen YJ, Shang CY, Tseng WYI, Gau SSF. Different neural substrates for executive functions in youths with ADHD: a diffusion spectrum imaging tractography study. Psychological medicine. 2016;46(6):1225-38.

21. Chiang H-L, Hsu Y-C, Shang C-Y, Tseng W-YI, Gau SS-F. White matter endophenotype candidates for ADHD: A diffusion imaging tractography study with sibling design. [References]. Psychological Medicine. 2020;50(7):1203-13.

22. Choi J, Lim, M. H., Lee, C., Park, J. K., Son, J., Shim, S. H., Yu, I. K., Khang, H. S., & Jeong, B. . Comparison of Diffusion Tensor Imaging in Attention Deficit Hyperactivity Disorder Boys with or without Comorbid Tic Disorders. Journal of Korean Neuropsychiatric Association. 2008;47(5):493–502.

23. Chuang T-C, Wu M-T, Huang S-P, Weng M-J, Yang P. Diffusion tensor imaging study of white matter fiber tracts in adolescent attention-deficit/hyperactivity disorder. [References]. Psychiatry Research: Neuroimaging. 2013;211(2):186-7.

24. Cooper M, Thapar A, Jones DK. White matter microstructure predicts autistic traits in attention-deficit/hyperactivity disorder. [References]. Journal of Autism and Developmental Disorders. 2014;44(11):2742-54.

25. Cooper M, Thapar A, Jones DK. ADHD severity is associated with white matter microstructure in the subgenual cingulum. NeuroImage Clinical. 2015;7:653-60.

26. Damatac CG, Chauvin RJM, Zwiers MP, van Rooij D, Akkermans SEA, Naaijen J, et al. White Matter Microstructure in Attention-Deficit/Hyperactivity Disorder: A Systematic Tractography Study in 654 Individuals. Biol Psychiatry Cogn Neurosci Neuroimaging. 2020.

27. Davenport ND, Karatekin C, White T, Lim KO. Differential fractional anisotropy abnormalities in adolescents with ADHD or schizophrenia. Psychiatry Research. 2010;181(3):193-8.

28. de Luis-Garcia R, Cabus-Pinol G, Imaz-Roncero C, Argibay-Quinones D, Barrio-Arranz G, Aja-Fernandez S, et al. Attention deficit/hyperactivity disorder and medication with stimulants in young children: a DTI study. Progress in Neuro Psychopharmacology & Biological Psychiatry. 2015;57:176-84.

29. De Zeeuw P, Mandl RC, Hulshoff Pol HE, Van Engeland H, Durston S. Decreased frontostriatal microstructural organization in attention deficit/hyperactivity disorder. Human Brain Mapping. 2012;33(8):1941-51.

30. de Zeeuw P, Schnack HG, van Belle J, Weusten J, van Dijk S, Langen M, et al. Differential brain development with low and high IQ in attention-deficit/hyperactivity disorder. PLoS ONE [Electronic Resource]. 2012;7(4).

31. Douglas PK, Gutman B, Anderson A, Larios C, Lawrence KE, Narr K, et al. Hemispheric brain asymmetry differences in youths with attention-deficit/hyperactivity disorder. Neuroimage Clin. 2018;18:744-52.

32. Ercan ES, Suren S, Bacanli A, Yazici KU, Calli C, Ardic UA, et al. Altered structural connectivity is related to attention deficit/hyperactivity subtypes: A DTI study. Psychiatry Research: Neuroimaging. 2016;256:57-64.

33. Fall S, Querne L, Le Moing A-G, Berquin P. Individual differences in subcortical microstructure organization reflect reaction time performances during a flanker task: A diffusion tensor imaging study in children with and without ADHD. [References]. Psychiatry Research: Neuroimaging. 2015;233(1):50-6.

34. Fayed N, Modrego PJ, Castillo J, Davila J. Evidence of Brain Dysfunction in Attention Deficit-Hyperactivity Disorder: a Controlled Study with Proton Magnetic Resonance Spectroscopy. Academic Radiology. 2007;14(9):1029-35.

35. Francx W, Zwiers MP, Mennes M, Oosterlaan J, Heslenfeld D, Hoekstra PJ, et al. White matter microstructure and developmental improvement of hyperactive/impulsive symptoms in attention-deficit/hyperactivity disorder. Journal of child psychology and psychiatry, and allied disciplines. 2015;56(12):1289-97.

36. Francx W, Llera A, Mennes M, Zwiers MP, Faraone SV, Oosterlaan J, et al. Integrated analysis of gray and white matter alterations in attention-deficit/hyperactivity disorder. NeuroImage Clinical. 2016;11:357-67.

37. Fuelscher I, Hyde C, Anderson V, Silk TJ. White matter tract signatures of fiber density and morphology in ADHD. Cortex. 2021;138:329-40.

38. Gau SS, Tseng WL, Tseng WY, Wu YH, Lo YC. Association between microstructural integrity of frontostriatal tracts and school functioning: ADHD symptoms and executive function as mediators. Psychological Medicine. 2015;45(3):529-43.

39. Graziano PA, Garic D, Dick AS. Individual differences in white matter of the uncinate fasciculus and inferior fronto-occipital fasciculus: possible early biomarkers for callous-unemotional behaviors in young children with disruptive behavior problems. J Child Psychol Psychiatry. 2022;63(1):19-33.

40. Griffiths KR, Braund TA, Kohn MR, Clarke S, Williams LM, Korgaonkar MS. Structural brain network topology underpinning ADHD and response to methylphenidate treatment. Transl Psychiatry. 2021;11(1):150.

41. Hamilton LS, Levitt JG, O'Neill J, Alger JR, Luders E, Phillips OR, et al. Reduced white matter integrity in attention-deficit hyperactivity disorder. NeuroReport. 2008;19(17):1705-8.

42. Helpern JA, Adisetiyo V, Falangola MF, Hu C, Di Martino A, Williams K, et al. Preliminary evidence of altered gray and white matter microstructural development in the frontal lobe of adolescents with attention-deficit hyperactivity disorder: a diffusional kurtosis imaging study. Journal of Magnetic Resonance Imaging. 2011;33(1):17-23.

43. Hong S-B, Zalesky A, Fornito A, Park S, Yang Y-H, Park M-H, et al. Connectomic disturbances in attention-deficit/hyperactivity disorder: A whole-brain tractography analysis. [References]. Biological Psychiatry. 2014;76(8):656-63.

44. Hyde C, Fuelscher I, Sciberras E, Efron D, Anderson VA, Silk T. Understanding motor difficulties in children with ADHD: A fixel-based analysis of the corticospinal tract. Prog Neuropsychopharmacol Biol Psychiatry. 2021;105:110125.

45. Hyde C, Sciberras E, Efron D, Fuelscher I, Silk T. Reduced fine motor competence in children with ADHD is associated with atypical microstructural organization within the superior longitudinal fasciculus. Brain Imaging Behav. 2021;15(2):727-37.

46. Jacobson LA, Peterson DJ, Rosch KS, Crocetti D, Mori S, Mostofsky SH. Sex-Based Dissociation of White Matter Microstructure in Children With Attention-Deficit/Hyperactivity Disorder. Journal of the American Academy of Child & Adolescent Psychiatry. 2015;54(11):938-46.

47. King JB, Yurgelun-Todd D, Stoeckel A, Di Muzio JM, Lopez-Larson MP. Sex differences in white matter integrity in youths with attention-deficit/hyperactivity disorder: A pilot study. Frontiers in Neuroscience. 2015;9(JUL).

48. Kobel M, Bechtel N, Specht K, Klarhofer M, Weber P, Scheffler K, et al. Structural and functional imaging approaches in attention deficit/hyperactivity disorder: Does the temporal lobe play a key role? [References]. Psychiatry Research: Neuroimaging. 2010;183(3):230-6.

49. Langevin LM, Macmaster FP, Crawford S, Lebel C, Dewey D. Common white matter microstructure alterations in pediatric motor and attention disorders. Journal of Pediatrics. 2014;164(5):1157-64.e1.

50. Lawrence KE, Levitt JG, Loo SK, Ly R, Yee V, O'Neill J, et al. White matter microstructure in subjects with attention-deficit/hyperactivity disorder and their siblings. Journal of the American Academy of Child & Adolescent Psychiatry. 2013;52(4):431-40.

51. Lee S JB, Go HJ, Yang PS, Kwon MJ, Choi J. Occipito-Temporal Connectivity in Medication-Naïve ADHD Children:Preliminary Diffusion Tensor Imaging Study. J Korean Neuropsychiatr Assoc. 2009.

52. Lee D, Quattrocki Knight E, Song H, Lee S, Pae C, Yoo S, et al. Differential structure-function network coupling in the inattentive and combined types of attention deficit hyperactivity disorder. PLoS One. 2021;16(12):e0260295.

53. Lei D, Ma J, Du X, Shen G, Jin X, Gong Q. Microstructural abnormalities in the combined and inattentive subtypes of attention deficit hyperactivity disorder: a diffusion tensor imaging study. Scientific reports. 2014;4:6875.

54. Li QQ, Sun JH, Guo LT, Zang YF, Feng ZZ, Huang XQ, et al. Increased Fractional Anisotropy in White Matter of the Right Frontal Region in Children with Attention-Deficit/Hyperactivity Disorder: A Diffusion Tensor Imaging Study. Act Nerv Super Rediviva. 2010;52(3):193-9.

55. Lin HY, Gau SSF, Huang-Gu SL, Shang CY, Wu YH, Tseng WYI. Neural substrates of behavioral variability in attention deficit hyperactivity disorder: based on ex-Gaussian reaction time distribution and diffusion spectrum imaging tractography. Psychological medicine. 2014;44(8):1751-64.

56. Lin Q, Bu X, Wang M, Liang Y, Chen H, Wang W, et al. Aberrant white matter properties of the callosal tracts implicated in girls with attention-deficit/hyperactivity disorder. Brain Imaging Behav. 2020;14(3):728-35.

57. Lin Q, Bu X, Chen H, Liang Y, Wang W, Yi Y, et al. Sex differences in microstructural alterations in the corpus callosum tracts in drug-naïve children with ADHD. Brain Imaging Behav. 2022.

58. Malisza KL, Buss JL, Bolster RB, de Gervai PD, Woods-Frohlich L, Summers R, et al. Comparison of spatial working memory in children with prenatal alcohol exposure and those diagnosed with ADHD; A functional magnetic resonance imaging study. [References]. Journal of Neurodevelopmental Disorders. 2012;4(1):1-20.

59. Mazzetti C, Gonzales Damatac C, Sprooten E, Ter Huurne N, Buitelaar JK, Jensen O. Dorsal-to-ventral imbalance in the superior longitudinal fasciculus mediates methylphenidate's effect on beta oscillations in ADHD. Psychophysiology. 2022:e14008.

60. Nagel BJ, Bathula D, Herting M, Schmitt C, Kroenke CD, Fair D, et al. Altered white matter microstructure in children with attention-deficit/hyperactivity disorder. Journal of the American Academy of Child & Adolescent Psychiatry. 2011;50(3):283-92.

61. O'Conaill CR, Malisza KL, Buss JL, Bolster RB, Clancy C, De Gervai PD, et al. Visual search for feature conjunctions: An fMRI study comparing alcohol-related neurodevelopmental disorder (ARND) to ADHD. Journal of Neurodevelopmental Disorders. 2015;7(1).

62. O'Neill J, O'Connor MJ, Yee V, Ly R, Narr K, Alger JR, et al. Differential neuroimaging indices in prefrontal white matter in prenatal alcohol-associated ADHD versus idiopathic ADHD. Birth Defects Res. 2019;111(12):797-811.

63. O'Neill J, O'Connor MJ, Kalender G, Ly R, Ng A, Dillon A, et al. Combining neuroimaging and behavior to discriminate children with attention deficit-hyperactivity disorder with and without prenatal alcohol exposure. Brain Imaging Behav. 2022;16(1):69-77.

64. Park S, Lee J-M, Kim J-W, Kwon H, Cho S-C, Han DH, et al. Increased white matter connectivity in traumatized children with attention deficit hyperactivity disorder. [References]. Psychiatry Research: Neuroimaging. 2016;247:57-63.

65. Pastura G, Doering T, Gasparetto EL, Mattos P, Araújo AP. Exploratory analysis of diffusion tensor imaging in children with attention deficit hyperactivity disorder: evidence of abnormal white matter structure. Atten Defic Hyperact Disord. 2016;8(2):65-71.

66. Pavuluri MN, Yang S, Kamineni K, Passarotti AM, Srinivasan G, Harral EM, et al. Diffusion tensor imaging study of white matter fiber tracts in pediatric bipolar disorder and attention-deficit/hyperactivity disorder. [References]. Biological Psychiatry. 2009;65(7):586-93.

67. Peterson DJ, Ryan M, Rimrodt SL, Cutting LE, Denckla MB, Kaufmann WE, et al. Increased regional fractional anisotropy in highly screened attention-deficit hyperactivity disorder (ADHD). Journal of Child Neurology. 2011;26(10):1296-302.

68. Qian L, Li Y, Wang Y, Wang Y, Cheng X, Li CY, et al. Shared and Distinct Topologically Structural Connectivity Patterns in Autism Spectrum Disorder and Attention-Deficit/Hyperactivity Disorder. Frontiers in Neuroscience. 2021;15:13.

69. Qiu M-g, Ye Z, Li Q-y, Liu G-j, Xie B, Wang J. Changes of brain structure and function in ADHD children. [References]. Brain Topography. 2011;24(3-4):243-52.

70. Ray S, Miller M, Karalunas S, Robertson C, Grayson DS, Cary RP, et al. Structural and functional connectivity of the human brain in autism spectrum disorders and attention-deficit/hyperactivity disorder: A rich club-organization study. [References]. Human Brain Mapping. 2014;35(12):6032-48.

71. Rossi ASU, de Moura LM, de Mello CB, de Souza AAL, Muszkat M, Bueno OFA. Attentional profiles and white matter correlates in attention-deficit/hyperactivity disorder predominantly inattentive type. [References]. Frontiers in Psychiatry. 2015;6:122.

72. Saad JF, Griffiths KR, Kohn MR, Braund TA, Clarke S, Williams LM, et al. No support for white matter connectivity differences in the combined and inattentive ADHD presentations. PLoS One. 2021;16(5):e0245028.

73. Saenz AA, Villemonteix T, Slama H, Baijot S, Mary A, Baleriaux D, et al. Relationship Between White Matter Abnormalities and Neuropsychological Measures in Children With ADHD. Journal of Attention Disorders. 2020;24(7):1020-31.

74. Shang CY, Wu YH, Gau SS, Tseng WY. Disturbed microstructural integrity of the frontostriatal fiber pathways and executive dysfunction in children with attention deficit hyperactivity disorder. Psychological medicine. 2013;43(5):1093-107.

75. Silk TJ, Vance A, Rinehart N, Bradshaw JL, Cunnington R. Structural development of the basal ganglia in attention deficit hyperactivity disorder: a diffusion tensor imaging study. Psychiatry Research. 2009;172(3):220-5.

76. Silk TJ, Vance A, Rinehart N, Bradshaw JL, Cunnington R. White-matter abnormalities in attention deficit hyperactivity disorder: A diffusion tensor imaging study. [References]. Human Brain Mapping. 2009;30(9):2757-65.

77. Silk TJ, Vilgis V, Adamson C, Chen J, Smit L, Vance A, et al. Abnormal asymmetry in frontostriatal white matter in children with attention deficit hyperactivity disorder. [References]. Brain Imaging and Behavior. 2016;10(4):1080-9.

78. Stephens K, Silk TJ, Anderson V, Hazell P, Enticott PG, Sciberras E. Associations Between Limbic System White Matter Structure and Socio-Emotional Functioning in Children with ADHD + ASD. J Autism Dev Disord. 2021;51(8):2663-72.

79. Sun H, Chen Y, Huang Q, Lui S, Huang X, Shi Y, et al. Psychoradiologic Utility of MR Imaging for Diagnosis of Attention Deficit Hyperactivity Disorder: A Radiomics Analysis. Radiology. 2018;287(2):620-30.

80. Svatkova A, Nestrasil I, Rudser K, Fine JG, Bledsoe J, Semrud-Clikeman M. Unique white matter microstructural patterns in ADHD presentations-A diffusion tensor imaging study. [References]. Human Brain Mapping. 2016;37(9):3323-36.

81. Tamm L, Barnea-Goraly N, Reiss AL. Diffusion tensor imaging reveals white matter abnormalities in Attention-Deficit/Hyperactivity Disorder. Psychiatry Research. 2012;202(2):150-4.

82. Tremblay LK, Hammill C, Ameis SH, Bhaijiwala M, Mabbott DJ, Anagnostou E, et al. Tracking Inhibitory Control in Youth With ADHD: A Multi-Modal Neuroimaging Approach. Front Psychiatry. 2020;11:00831.

83. Tsai CJ, Lin HY, Tseng IW, Gau SS. White matter microstructural integrity correlates of emotion dysregulation in children with ADHD: A diffusion imaging tractography study. Prog Neuropsychopharmacol Biol Psychiatry. 2021;110:110325.

84. Unsel-Bolat G, Baytunca MB, Kardas B, Ipci M, Inci Izmir SB, Ozyurt O, et al. Diffusion tensor imaging findings in children with sluggish cognitive tempo comorbid Attention Deficit Hyperactivity Disorder. Nordic Journal of Psychiatry. 2020;74(8):620-6.

85. Wang L, Fan GG, Yu B, Ma HW, Pan XX, Guo QY. Correlated study between reaction time and fractional anisotropy for ADHD children. [Chinese]. Chinese Journal of Medical Imaging Technology. 2008;24(4):489-92.

86. Wang P, Jiang X, Chen H, Zhang S, Li X, Cao Q, et al. Assessing Fine-Granularity Structural and Functional Connectivity in Children With Attention Deficit Hyperactivity Disorder. Front Hum Neurosci. 2020;14:594830.

87. Wu Y-H, Gau SS-F, Lo Y-C, Tseng W-YI. White matter tract integrity of frontostriatal circuit in attention deficit hyperactivity disorder: Association with attention performance and symptoms. [References]. Human Brain Mapping. 2014;35(1):199-212.

88. Wu Z-M, Bralten J, Cao Q-J, Hoogman M, Zwiers MP, An L, et al. White matter microstructural alterations in children with ADHD: Categorical and dimensional perspectives. [References]. Neuropsychopharmacology. 2017;42(2):572-80.

89. Wu ZM, Llera A, Hoogman M, Cao QJ, Zwiers MP, Bralten J, et al. Linked anatomical and functional brain alterations in children with attention-deficit/hyperactivity disorder. Neuroimage Clin. 2019;23:101851.

90. Wu W, McAnulty G, Hamoda HM, Sarill K, Karmacharya S, Gagoski B, et al. Detecting microstructural white matter abnormalities of frontal pathways in children with ADHD using advanced diffusion models. Brain Imaging Behav. 2020;14(4):981-97.

91. Wu ZM, Wang P, Yang L, Liu L, Sun L, An L, et al. Altered brain white matter microstructural asymmetry in children with ADHD. Psychiatry Res. 2020;285:112817.

92. Wu ZM, Wang P, Liu L, Liu J, Cao XL, Sun L, et al. ADHD-inattentive versus ADHD-Combined subtypes: A severity continuum or two distinct entities? A comprehensive analysis of clinical, cognitive and neuroimaging data. J Psychiatr Res. 2022;149:28-36.

93. Xia S, Li X, Kimball AE, Kelly MS, Lesser I, Branch C. Thalamic shape and connectivity abnormalities in children with attention-deficit/hyperactivity disorder. Psychiatry Research. 2012;204(2-3):161-7.

94. Yoo JH, Kim JI, Kim BN, Jeong B. Exploring characteristic features of attention-deficit/hyperactivity disorder: findings from multi-modal MRI and candidate genetic data. Brain Imaging and Behavior. 2020;14(6):2132-47.

95. Zhan C, Liu Y, Wu K, Gao Y, Li X. Structural and Functional Abnormalities in Children with Attention-Deficit/Hyperactivity Disorder: A Focus on Subgenual Anterior Cingulate Cortex. Brain Connect. 2017;7(2):106-14.

96. Zhou X, Lin Q, Gui Y, Wang Z, Liu M, Lu H. Multimodal MR Images-Based Diagnosis of Early Adolescent Attention-Deficit/Hyperactivity Disorder Using Multiple Kernel Learning. Front Neurosci. 2021;15:710133.

97. Bode MK, Lindholm P, Kiviniemi V, Moilanen I, Ebeling H, Veijola J, et al. DTI abnormalities in adults with past history of attention deficit hyperactivity disorder: a tract-based spatial statistics study. Acta Radiologica. 2015;56(8):990-6.

98. Bouziane C, Caan MWA, Tamminga HGH, Schrantee A, Bottelier MA, de Ruiter MB, et al. ADHD and maturation of brain white matter: A DTI study in medication naive children and adults. Neuroimage Clin. 2018;17:53-9.

99. Chaim TM, Zhang T, Zanetti MV, Da Silva MA, Louza MR, Doshi J, et al. Multimodal magnetic resonance imaging study of Treatment-Naive Adults with Attention-Deficit/Hyperactivity Disorder. PLoS ONE. 2014;9(10).

100. Chaim-Avancini TM, Doshi J, Zanetti MV, Erus G, Silva MA, Duran FLS, et al. Neurobiological support to the diagnosis of ADHD in stimulant-naïve adults: pattern recognition analyses of MRI data. Acta Psychiatr Scand. 2017;136(6):623-36.

101. Chiang HL, Chen YJ, Lin HY, Tseng WI, Gau SS. Disorder-Specific Alteration in White Matter Structural Property in Adults With Autism Spectrum Disorder Relative to Adults With ADHD and Adult Controls. Hum Brain Mapp. 2017;38(1):384-95.

102. Chiang HL, Yang LK, Chen YJ, Hsu YC, Lo YC, Tseng WYI, et al. Altered White-matter Tract Property in Adults with Attention-deficit Hyperactivity Disorder. Neuroscience. 2022;487:78-87.

103. Cortese S, Imperati D, Zhou J, Proal E, Klein RG, Mannuzza S, et al. White matter alterations at 33-year follow-up in adults with childhood attention-deficit/hyperactivity disorder. Biological Psychiatry. 2013;74(8):591-8.

104. Dramsdahl M, Westerhausen R, Haavik J, Hugdahl K, Plessen KJ. Adults with attention-deficit/hyperactivity disorder - a diffusion-tensor imaging study of the corpus callosum. Psychiatry Research. 2012;201(2):168-73.

105. Elliott BL, D'Ardenne K, Mukherjee P, Schweitzer JB, McClure SM. Limbic and Executive Meso- and Nigro-striatal Tracts Predict Impulsivity Differences in ADHD. Biol Psychiatry Cogn Neurosci Neuroimaging. 2021.

106. Gehricke JG, Kruggel F, Thampipop T, Alejo SD, Tatos E, Fallon J, et al. The brain anatomy of attention-deficit/hyperactivity disorder in young adults - a magnetic resonance imaging study. PLoS One. 2017;12(4):e0175433.

107. Hearne LJ, Lin H-Y, Sanz-Leon P, Tseng W-YI, Gau SS-F, Roberts JA, et al. Adhd symptoms map onto noise-driven structure-function decoupling between hub and peripheral brain regions. [References]. Molecular Psychiatry. 2019(Pagination).

108. Kölle M, Mackert S, Heckel K, Philipsen A, Ulrich M, Grön G. Lower fractional anisotropy of the corticothalamic tract and increased response time variability in adult patients with ADHD. J Psychiatry Neurosci. 2022;47(2):E99-e108.

109. Konrad A, Dielentheis TF, El Masri D, Bayerl M, Fehr C, Gesierich T, et al. Disturbed structural connectivity is related to inattention and impulsivity in adult attention deficit hyperactivity disorder. European Journal of Neuroscience. 2010;31(5):912-9.

110. Konrad A, Dielentheis TF, El Masri D, Dellani PR, Stoeter P, Vucurevic G, et al. White matter abnormalities and their impact on attentional performance in adult attention-deficit/hyperactivity disorder. European Archives of Psychiatry & Clinical Neuroscience. 2012;262(4):351-60.

111. Li D, Li T, Niu Y, Xiang J, Cao R, Liu B, et al. Reduced hemispheric asymmetry of brain anatomical networks in attention deficit hyperactivity disorder. Brain Imaging Behav. 2019;13(3):669-84.

112. Li D, Cui X, Yan T, Liu B, Zhang H, Xiang J, et al. Abnormal Rich Club Organization in Hemispheric White Matter Networks of ADHD. J Atten Disord. 2021;25(9):1215-29.

113. Luo YY, Halperin JM, Li XB. Anatomical substrates of symptom remission and persistence in young adults with childhood attention deficit/hyperactivity disorder. European Neuropsychopharmacology. 2020;33:117-25.

114. Luo Y, Alvarez TL, Halperin JM, Li X. Multimodal neuroimaging-based prediction of adult outcomes in childhood-onset ADHD using ensemble learning techniques. Neuroimage Clin. 2020;26:102238.

115. Makris N, Buka SL, Biederman J, Papadimitriou GM, Hodge SM, Valera EM, et al. Attention and executive systems abnormalities in adults with childhood adhd: A DT-MRI study of connections. Cerebral Cortex. 2008;18(5):1210-20.

116. Ohta H, Aoki YY, Itahashi T, Kanai C, Fujino J, Nakamura M, et al. White matter alterations in autism spectrum disorder and attention-deficit/hyperactivity disorder in relation to sensory profile. Mol Autism. 2020;11(1):13.

117. Onnink AM, Zwiers MP, Hoogman M, Mostert JC, Dammers J, Kan CC, et al. Deviant white matter structure in adults with attention-deficit/hyperactivity disorder points to aberrant myelination and affects neuropsychological performance. Progress in Neuro Psychopharmacology & Biological Psychiatry. 2015;63:14-22.

118. Schweren LJ, Hartman CA, Zwiers MP, Heslenfeld DJ, Franke B, Oosterlaan J, et al. Stimulant treatment history predicts frontal-striatal structural connectivity in adolescents with attention-deficit/hyperactivity disorder. Eur Neuropsychopharmacol. 2016;26(4):674-83.

119. Shaw P, Sudre G, Wharton A, Weingart D, Sharp W, Sarlls J. White matter microstructure and the variable adult outcome of childhood attention deficit hyperactivity disorder. Neuropsychopharmacology. 2015;40(3):746-54.

120. Sidlauskaite J, Caeyenberghs K, Sonuga-Barke E, Roeyers H, Wiersema JR. Whole-brain structural topology in adult attention-deficit/hyperactivity disorder: Preserved global - disturbed local network organization. NeuroImage Clinical. 2015;9:506-12.

121. Tung YH, Lin HY, Chen CL, Shang CY, Yang LY, Hsu YC, et al. Whole Brain White Matter Tract Deviation and Idiosyncrasy From Normative Development in Autism and ADHD and Unaffected Siblings Link With Dimensions of Psychopathology and Cognition. Am J Psychiatry. 2021:appiajp202020070999.

122. van Ewijk H, Heslenfeld DJ, Zwiers MP, Faraone SV, Luman M, Hartman CA, et al. Different mechanisms of white matter abnormalities in attention-deficit/hyperactivity disorder: a diffusion tensor imaging study. Journal of the American Academy of Child & Adolescent Psychiatry. 2014;53(7):790-9.

123. Van Ewijk H, Groenman AP, Zwiers MP, Heslenfeld DJ, Faraone SV, Hartman CA, et al. Smoking and the developing brain: Altered white matter microstructure in attention-deficit/hyperactivity disorder and healthy controls. Human Brain Mapping. 2015;36(3):1180-9.

124. van Ewijk H, Bralten J, van Duin EDA, Hakobjan M, Buitelaar JK, Heslenfeld DJ, et al. Female-specific association of NOS1 genotype with white matter microstructure in ADHD patients and controls. J Child Psychol Psychiatry. 2017;58(8):958-66.

125. Versace A, Jones NP, Joseph HM, Lindstrom RA, Wilson TK, Lima Santos JP, et al. White matter abnormalities associated with ADHD outcomes in adulthood. Mol Psychiatry. 2021.

126. Wang B, Wang GS, Wang X, Cao R, Xiang J, Yan T, et al. Rich-Club Analysis in Adults With ADHD Connectomes Reveals an Abnormal Structural Core Network. Journal of Attention Disorders. 2021;25(8):1068-79.

127. Wolfers T, Onnink AM, Zwiers MP, Arias-Vasquez A, Hoogman M, Mostert JC, et al. Lower white matter microstructure in the superior longitudinal fasciculus is associated with increased response time variability in adults with attention-deficit/ hyperactivity disorder. Journal of Psychiatry & Neuroscience. 2015;40(5):344-51.

128. Wolfers T, Arenas AL, Onnink AMH, Dammers J, Hoogman M, Zwiers MP, et al. Refinement by integration: aggregated effects of multimodal imaging markers on adult ADHD. J Psychiatry Neurosci. 2017;42(6):386-94.

129. Yoncheva YN, Somandepalli K, Reiss PT, Kelly C, Di Martino A, Lazar M, et al. Mode of Anisotropy Reveals Global Diffusion Alterations in Attention-Deficit/Hyperactivity Disorder. Journal of the American Academy of Child & Adolescent Psychiatry. 2016;55(2):137-45.

**REFERENCES IN SUPPLEMENTARY MATERIAL**

1. Albaugh MD, Hudziak JJ, Ing A, Chaarani B, Barker E, Jia TY, et al. White matter microstructure is associated with hyperactive/inattentive symptomatology and polygenic risk for attention-deficit/hyperactivity disorder in a population-based sample of adolescents. Neuropsychopharmacology. 2019;44(9):1597-603.

2. Bouziane C, Filatova OG, Schrantee A, Caan MWA, Vos FM, Reneman L. White Matter by Diffusion MRI Following Methylphenidate Treatment: A Randomized Control Trial in Males with Attention-Deficit/Hyperactivity Disorder. Radiology. 2019;293(1):186-92.

3. Casey BJ, Epstein JN, Buhle J, Liston C, Davidson MC, Tonev ST, et al. Frontostriatal connectivity and its role in cognitive control in parent-child dyads with ADHD. American Journal of Psychiatry. 2007;164(11):1729-36.

4. Forde NJ, Naaijen J, Lythgoe DJ, Akkermans SEA, Openneer TJC, Dietrich A, et al. Multi-modal imaging investigation of anterior cingulate cortex cytoarchitecture in neurodevelopment. European Neuropsychopharmacology. 2018;28(1):13-23.

5. Fu G-H, Chen W, Li H-M, Wang Y-F, Liu L, Qian Q-J. A potential association of RNF219-AS1 with ADHD: Evidence from categorical analysis of clinical phenotypes and from quantitative exploration of executive function and white matter microstructure endophenotypes. [References]. CNS Neuroscience & Therapeutics. 2021;27(5):603-16.

6. Hong SB, Zalesky A, Park S, Yang YH, Park MH, Kim B, et al. COMT genotype affects brain white matter pathways in attention-deficit/hyperactivity disorder. Human Brain Mapping. 2015;36(1):367-77.

7. Ing A, Samann PG, Chu C, Tay N, Biondo F, Robert G, et al. Identification of neurobehavioural symptom groups based on shared brain mechanisms. Nature Human Behaviour. 1306;3(12):1306-18.

8. Jacobs GR, Voineskos AN, Hawco C, Stefanik L, Forde NJ, Dickie EW, et al. Integration of brain and behavior measures for identification of data-driven groups cutting across children with asd, adhd, or ocd. [References]. Neuropsychopharmacology. 2020(Pagination).

9. McNally MA, Crocetti D, Mahone EM, Denckla MB, Suskauer SJ, Mostofsky SH. Corpus callosum segment circumference is associated with response control in children with attention-deficit hyperactivity disorder (ADHD). Journal of Child Neurology. 2010;25(4):453-62.

10. Park S, Hong SB, Kim JW, Yang YH, Park MH, Kim BN, et al. White-matter connectivity and methylphenidate-induced changes in attentional performance according to alpha2A-adrenergic receptor gene polymorphisms in Korean children with attention-deficit hyperactivity disorder. Journal of Neuropsychiatry & Clinical Neurosciences. 2013;25(3):222-8.

11. Poldrack RA, Congdon E, Triplett W, Gorgolewski KJ, Karlsgodt KH, Mumford JA, et al. A phenome-wide examination of neural and cognitive function. Sci Data. 2016;3:160110.

12. Rossi ASU, Moura LM, Miranda MC, Muszkat M, Mello CB, Bueno OFA. Latent class analysis of attention and white matter correlation in children with attention-deficit/hyperactivity disorder. Braz J Med Biol Res. 2018;51(11):e7653.

13. Sudre G, Bouyssi-Kobar M, Norman L, Sharp W, Choudhury S, Shaw P. Estimating the Heritability of Developmental Change in Neural Connectivity, and Its Association With Changing Symptoms of Attention-Deficit/Hyperactivity Disorder. Biol Psychiatry. 2021;89(5):443-50.

14. Sudre G, Choudhuri S, Szekely E, Bonner T, Goduni E, Sharp W, et al. Estimating the Heritability of Structural and Functional Brain Connectivity in Families Affected by Attention-Deficit/Hyperactivity Disorder. JAMA Psychiatry. 2017;74(1):76-84.

15. van Ewijk H, Noordermeer SD, Heslenfeld DJ, Luman M, Hartman CA, Hoekstra PJ, et al. The influence of comorbid oppositional defiant disorder on white matter microstructure in attention-deficit/hyperactivity disorder. Eur Child Adolesc Psychiatry. 2016;25(7):701-10.

16. van Schouwenburg MR, Onnink AM, ter Huurne N, Kan CC, Zwiers MP, Hoogman M, et al. Cognitive flexibility depends on white matter microstructure of the basal ganglia. Neuropsychologia. 2014;53:171-7.

17. Damatac CG, Soheili-Nezhad S, Freches GB, Zwiers MP, de Bruijn S, Ikde S, et al. Longitudinal changes of ADHD symptoms in association with white matter microstructure: a tract-specific fixel-based analysis. bioRxiv. 2021;19.

18. Ameis SH. Heterogeneity within and between autism spectrum disorder and attention-deficit/hyperactivity disorder challenge or opportunity? JAMA Psychiatry. 2017;74(11):1093-4.

19. Ameis SH. Distinct and Shared White Matter Disruption and Dimensional Brain-Behavior Relationships in Children With ASD, ADHD, and OCD Compared to Children Without Mental Health Diagnoses. Journal of the American Academy of Child and Adolescent Psychiatry. 2018;57 (10 Supplement):S294.

20. Chaim TM, Silva MA, Varol E, Doshi J, Zanetti MV, Gaonkar B, et al. High-dimensional pattern classification of brain morphometric and dti data of adult ADHD. Biological Psychiatry. 2012;71(8):190S.

21. Clerkin SM, Tang CY, Halperin JM. Thalamocortical and frontostriatal circuits in attention-deficit/hyperactivity disorder: A functional magnetic resonance and diffusion tensor imaging study. Clinical and Translational Science. 2010;3 (2):S38.

22. De Zeeuw P, Mandl RCW, Hulshoff Pol HE, Van Engeland H, Durston S. Decreased fronto-striatal structural connectivity in ADHD. Neuropsychopharmacology. 2010;35:S263.

23. Hoogman M. Structural Connectivity in ADHD: Evidence From 2500 Individuals From the ENIGMA-ADHD Collaboration. Biological Psychiatry. 2020;87 (9 Supplement):S87.

24. Li S, Qingjiu C, Yufeng Z, Manqiu S, Gaolang G, Yufeng W. A diffusion tensor imaging study of the anterior cingulum white matter in children with attention-deficit/hyperactivity disorder. J Neural Transm. 2007;114(7):LXXII-LXXII.

25. Rossi A, Hermann V, Alves MV, Mello C, Bueno O. Emotional recognition and white matter abnormalities in ADHD-I. ADHD Attention Deficit and Hyperactivity Disorders. 2015;7:S35.

26. Rubia K. White Matter Structure and Delay Tolerance in Attention-Deficit/Hyperactivity Disorder. Biological Psychiatry : Cognitive Neuroscience and Neuroimaging. 2019;4(3):213-5.

27. Schweren L, Hartman C, Zwiers M, Heslenfeld D, Oosterlaan J, Franke B, et al. Stimulant treatment history predicts frontal-striatal structural connectivity in adolescents with attentiondeficit/hyperactivity disorder. European Child and Adolescent Psychiatry. 2015;24(1):S109.

28. Shaw P, Sudre G. Estimating the heritability of the brain's structural connectivity and its association with changing symptoms of attention deficit hyperactivity disorder. Neuropsychopharmacology. 2019;44 (Supplement 1):413-4.

29. Posner J. A neuroscience perspective on the heterogeneity of attention-deficit/ hyperactivity disorder. Journal of the American Academy of Child and Adolescent Psychiatry. 2016;55 (10 Supplement 1):S328-S9.

30. Park MH, Park S, Kim HJ, Kim JW, Shin MS, Kim BN, et al. The associations between alpha-2A-adrenergic receptor gene polymorphism, neuropsychological performance and white matter connectivity among ADHD children. European Child and Adolescent Psychiatry. 2011;20:S124-S5.

31. Smullen D. A DWI study investigating the association between structural connectivity of the frontoparietal network with response inhibition difficulties in ADHD. Brain and Neuroscience Advances. 2021;5:222-3.

32. Cupertino RB, Soheili-Nezhad S, Grevet EH, Bandeira CE, Picon FA, Tavares MEA, et al. Reduced fronto-striatal volume in attention-deficit/hyperactivity disorder in two cohorts across the lifespan. Neuroimage Clin. 2020;28:102403.

33. He HG, Wen HW, Dai D, Wang JQ. Computer-Aided Prognosis: Accurate Prediction of Patients with Neurologic and Psychiatric Diseases via Multi-modal MRI Analysis. In: Suzuki K, Chen Y, editors. Artificial Intelligence in Decision Support Systems for Diagnosis in Medical Imaging. Intelligent Systems Reference Library. 140. Berlin: Springer-Verlag Berlin; 2018. p. 225-65.

34. Öztekin I, Finlayson MA, Graziano PA, Dick AS. Is there any incremental benefit to conducting neuroimaging and neurocognitive assessments in the diagnosis of ADHD in young children? A machine learning investigation. Dev Cogn Neurosci. 2021;49:100966.

35. Ameis SH, Lerch JP, Taylor MJ, Lee W, Viviano JD, Pipitone J, et al. A diffusion tensor imaging study in children with ADHD, autism spectrum disorder, OCD, and matched controls: Distinct and non-distinct white matter disruption and dimensional brain-behavior relationships. [References]. The American Journal of Psychiatry. 2016;173(12):1213-22.

36. Ball G, Malpas CB, Genc S, Efron D, Sciberras E, Anderson V, et al. Multimodal Structural Neuroimaging Markers of Brain Development and ADHD Symptoms. American Journal of Psychiatry. 2019;176(1):57-66.

37. Basay BK, Buber A, Basay O, Alacam H, Ozturk O, Suren S, et al. White matter alterations related to attention-deficit hyperactivity disorder and COMT val158met polymorphism: Children with valine homozygote attention-deficit hyperactivity disorder have altered white matter connectivity in the right cingulum (cingulate gyrus). [References]. Neuropsychiatric Disease and Treatment. 2016;12:969-81.

38. Dramsdahl M, Westerhausen R, Haavik J, Hugdahl K, Plessen KJ. Adults with attention-deficit/hyperactivity disorder - a diffusion-tensor imaging study of the corpus callosum. Psychiatry Research. 2012;201(2):168-73.

39. Francx W, Zwiers MP, Mennes M, Oosterlaan J, Heslenfeld D, Hoekstra PJ, et al. White matter microstructure and developmental improvement of hyperactive/impulsive symptoms in attention-deficit/hyperactivity disorder. Journal of child psychology and psychiatry, and allied disciplines. 2015;56(12):1289-97.

40. Francx W, Llera A, Mennes M, Zwiers MP, Faraone SV, Oosterlaan J, et al. Integrated analysis of gray and white matter alterations in attention-deficit/hyperactivity disorder. NeuroImage Clinical. 2016;11:357-67.

41. O'Neill J, O'Connor MJ, Yee V, Ly R, Narr K, Alger JR, et al. Differential neuroimaging indices in prefrontal white matter in prenatal alcohol-associated ADHD versus idiopathic ADHD. Birth Defects Res. 2019;111(12):797-811.

42. Park S, Lee J-M, Kim J-W, Kwon H, Cho S-C, Han DH, et al. Increased white matter connectivity in traumatized children with attention deficit hyperactivity disorder. [References]. Psychiatry Research: Neuroimaging. 2016;247:57-63.

43. Pastura G, Doering T, Gasparetto EL, Mattos P, Araújo AP. Exploratory analysis of diffusion tensor imaging in children with attention deficit hyperactivity disorder: evidence of abnormal white matter structure. Atten Defic Hyperact Disord. 2016;8(2):65-71.

44. Saad JF, Griffiths KR, Kohn MR, Braund TA, Clarke S, Williams LM, et al. No support for white matter connectivity differences in the combined and inattentive ADHD presentations. PLoS One. 2021;16(5):e0245028.

45. Tamm L, Barnea-Goraly N, Reiss AL. Diffusion tensor imaging reveals white matter abnormalities in Attention-Deficit/Hyperactivity Disorder. Psychiatry Research. 2012;202(2):150-4.

46. Van Ewijk H, Groenman AP, Zwiers MP, Heslenfeld DJ, Faraone SV, Hartman CA, et al. Smoking and the developing brain: Altered white matter microstructure in attention-deficit/hyperactivity disorder and healthy controls. Human Brain Mapping. 2015;36(3):1180-9.

47. van Ewijk H, Bralten J, van Duin EDA, Hakobjan M, Buitelaar JK, Heslenfeld DJ, et al. Female-specific association of NOS1 genotype with white matter microstructure in ADHD patients and controls. J Child Psychol Psychiatry. 2017;58(8):958-66.

48. Wolfers T, Onnink AM, Zwiers MP, Arias-Vasquez A, Hoogman M, Mostert JC, et al. Lower white matter microstructure in the superior longitudinal fasciculus is associated with increased response time variability in adults with attention-deficit/ hyperactivity disorder. Journal of Psychiatry & Neuroscience. 2015;40(5):344-51.

49. Wolfers T, Arenas AL, Onnink AMH, Dammers J, Hoogman M, Zwiers MP, et al. Refinement by integration: aggregated effects of multimodal imaging markers on adult ADHD. J Psychiatry Neurosci. 2017;42(6):386-94.

50. Wu ZM, Llera A, Hoogman M, Cao QJ, Zwiers MP, Bralten J, et al. Linked anatomical and functional brain alterations in children with attention-deficit/hyperactivity disorder. Neuroimage Clin. 2019;23:101851.

51. Wu ZM, Wang P, Yang L, Liu L, Sun L, An L, et al. Altered brain white matter microstructural asymmetry in children with ADHD. Psychiatry Res. 2020;285:112817.

52. Yoo JH, Kim JI, Kim BN, Jeong B. Exploring characteristic features of attention-deficit/hyperactivity disorder: findings from multi-modal MRI and candidate genetic data. Brain Imaging and Behavior. 2020;14(6):2132-47.

53. Adisetiyo V, Tabesh A, Di Martino A, Falangola MF, Castellanos FX, Jensen JH, et al. Attention-deficit/hyperactivity disorder without comorbidity is associated with distinct atypical patterns of cerebral microstructural development. [References]. Human Brain Mapping. 2014;35(5):2148-62.

54. Aoki Y, Yoncheva YN, Chen B, Nath T, Sharp D, Lazar M, et al. Association of white matter structure with autism spectrum disorder and attention-deficit/hyperactivity disorder. [References]. JAMA Psychiatry. 2017;74(11):1120-8.

55. Bessette KL, Stevens MC. Neurocognitive Pathways in Attention-Deficit/Hyperactivity Disorder and White Matter Microstructure. Biol Psychiatry Cogn Neurosci Neuroimaging. 2019;4(3):233-42.

56. Bode MK, Lindholm P, Kiviniemi V, Moilanen I, Ebeling H, Veijola J, et al. DTI abnormalities in adults with past history of attention deficit hyperactivity disorder: a tract-based spatial statistics study. Acta Radiologica. 2015;56(8):990-6.

57. Bos DJ, Oranje B, Achterberg M, Vlaskamp C, Ambrosino S, de Reus MA, et al. Structural and functional connectivity in children and adolescents with and without attention deficit/hyperactivity disorder. J Child Psychol Psychiatry. 2017;58(7):810-8.

58. Bouziane C, Caan MWA, Tamminga HGH, Schrantee A, Bottelier MA, de Ruiter MB, et al. ADHD and maturation of brain white matter: A DTI study in medication naive children and adults. Neuroimage Clin. 2018;17:53-9.

59. Chuang T-C, Wu M-T, Huang S-P, Weng M-J, Yang P. Diffusion tensor imaging study of white matter fiber tracts in adolescent attention-deficit/hyperactivity disorder. [References]. Psychiatry Research: Neuroimaging. 2013;211(2):186-7.

60. Cooper M, Thapar A, Jones DK. White matter microstructure predicts autistic traits in attention-deficit/hyperactivity disorder. [References]. Journal of Autism and Developmental Disorders. 2014;44(11):2742-54.

61. Cortese S, Imperati D, Zhou J, Proal E, Klein RG, Mannuzza S, et al. White matter alterations at 33-year follow-up in adults with childhood attention-deficit/hyperactivity disorder. Biological Psychiatry. 2013;74(8):591-8.

62. de Luis-Garcia R, Cabus-Pinol G, Imaz-Roncero C, Argibay-Quinones D, Barrio-Arranz G, Aja-Fernandez S, et al. Attention deficit/hyperactivity disorder and medication with stimulants in young children: a DTI study. Progress in Neuro Psychopharmacology & Biological Psychiatry. 2015;57:176-84.

63. Ercan ES, Suren S, Bacanli A, Yazici KU, Calli C, Ardic UA, et al. Altered structural connectivity is related to attention deficit/hyperactivity subtypes: A DTI study. Psychiatry Research: Neuroimaging. 2016;256:57-64.

64. King JB, Yurgelun-Todd D, Stoeckel A, Di Muzio JM, Lopez-Larson MP. Sex differences in white matter integrity in youths with attention-deficit/hyperactivity disorder: A pilot study. Frontiers in Neuroscience. 2015;9(JUL).

65. Nagel BJ, Bathula D, Herting M, Schmitt C, Kroenke CD, Fair D, et al. Altered white matter microstructure in children with attention-deficit/hyperactivity disorder. Journal of the American Academy of Child & Adolescent Psychiatry. 2011;50(3):283-92.

66. O'Conaill CR, Malisza KL, Buss JL, Bolster RB, Clancy C, De Gervai PD, et al. Visual search for feature conjunctions: An fMRI study comparing alcohol-related neurodevelopmental disorder (ARND) to ADHD. Journal of Neurodevelopmental Disorders. 2015;7(1).

67. Ohta H, Aoki YY, Itahashi T, Kanai C, Fujino J, Nakamura M, et al. White matter alterations in autism spectrum disorder and attention-deficit/hyperactivity disorder in relation to sensory profile. Mol Autism. 2020;11(1):13.

68. Onnink AM, Zwiers MP, Hoogman M, Mostert JC, Dammers J, Kan CC, et al. Deviant white matter structure in adults with attention-deficit/hyperactivity disorder points to aberrant myelination and affects neuropsychological performance. Progress in Neuro Psychopharmacology & Biological Psychiatry. 2015;63:14-22.

69. Rossi ASU, de Moura LM, de Mello CB, de Souza AAL, Muszkat M, Bueno OFA. Attentional profiles and white matter correlates in attention-deficit/hyperactivity disorder predominantly inattentive type. [References]. Frontiers in Psychiatry. 2015;6:122.

70. Saenz AA, Villemonteix T, Slama H, Baijot S, Mary A, Baleriaux D, et al. Relationship Between White Matter Abnormalities and Neuropsychological Measures in Children With ADHD. Journal of Attention Disorders. 2020;24(7):1020-31.

71. Silk TJ, Vance A, Rinehart N, Bradshaw JL, Cunnington R. White-matter abnormalities in attention deficit hyperactivity disorder: A diffusion tensor imaging study. [References]. Human Brain Mapping. 2009;30(9):2757-65.

72. Svatkova A, Nestrasil I, Rudser K, Fine JG, Bledsoe J, Semrud-Clikeman M. Unique white matter microstructural patterns in ADHD presentations-A diffusion tensor imaging study. [References]. Human Brain Mapping. 2016;37(9):3323-36.

73. Unsel-Bolat G, Baytunca MB, Kardas B, Ipci M, Inci Izmir SB, Ozyurt O, et al. Diffusion tensor imaging findings in children with sluggish cognitive tempo comorbid Attention Deficit Hyperactivity Disorder. Nordic Journal of Psychiatry. 2020;74(8):620-6.

74. van Ewijk H, Heslenfeld DJ, Zwiers MP, Faraone SV, Luman M, Hartman CA, et al. Different mechanisms of white matter abnormalities in attention-deficit/hyperactivity disorder: a diffusion tensor imaging study. Journal of the American Academy of Child & Adolescent Psychiatry. 2014;53(7):790-9.

75. Wu Z-M, Bralten J, Cao Q-J, Hoogman M, Zwiers MP, An L, et al. White matter microstructural alterations in children with ADHD: Categorical and dimensional perspectives. [References]. Neuropsychopharmacology. 2017;42(2):572-80.

76. Wu ZM, Wang P, Liu L, Liu J, Cao XL, Sun L, et al. ADHD-inattentive versus ADHD-Combined subtypes: A severity continuum or two distinct entities? A comprehensive analysis of clinical, cognitive and neuroimaging data. J Psychiatr Res. 2022;149:28-36.

77. Yoncheva YN, Somandepalli K, Reiss PT, Kelly C, Di Martino A, Lazar M, et al. Mode of Anisotropy Reveals Global Diffusion Alterations in Attention-Deficit/Hyperactivity Disorder. Journal of the American Academy of Child & Adolescent Psychiatry. 2016;55(2):137-45.

78. Fall S, Querne L, Le Moing A-G, Berquin P. Individual differences in subcortical microstructure organization reflect reaction time performances during a flanker task: A diffusion tensor imaging study in children with and without ADHD. [References]. Psychiatry Research: Neuroimaging. 2015;233(1):50-6.

79. Hong S-B, Zalesky A, Fornito A, Park S, Yang Y-H, Park M-H, et al. Connectomic disturbances in attention-deficit/hyperactivity disorder: A whole-brain tractography analysis. [References]. Biological Psychiatry. 2014;76(8):656-63.

80. Jacobson LA, Peterson DJ, Rosch KS, Crocetti D, Mori S, Mostofsky SH. Sex-Based Dissociation of White Matter Microstructure in Children With Attention-Deficit/Hyperactivity Disorder. Journal of the American Academy of Child & Adolescent Psychiatry. 2015;54(11):938-46.

81. Lin HY, Gau SSF, Huang-Gu SL, Shang CY, Wu YH, Tseng WYI. Neural substrates of behavioral variability in attention deficit hyperactivity disorder: based on ex-Gaussian reaction time distribution and diffusion spectrum imaging tractography. Psychological medicine. 2014;44(8):1751-64.

82. Chiang H-L, Chen Y-J, Lo Y-C, Tseng W-YI, Gau SS-F. Altered white matter tract property related to impaired focused attention, sustained attention, cognitive impulsivity and vigilance in attention-deficit/ hyperactivity disorder. [References]. Journal of Psychiatry & Neuroscience. 2015;40(5):325-35.

83. Chiang HL, Chen YJ, Shang CY, Tseng WYI, Gau SSF. Different neural substrates for executive functions in youths with ADHD: a diffusion spectrum imaging tractography study. Psychological medicine. 2016;46(6):1225-38.

84. Gau SS, Tseng WL, Tseng WY, Wu YH, Lo YC. Association between microstructural integrity of frontostriatal tracts and school functioning: ADHD symptoms and executive function as mediators. Psychological Medicine. 2015;45(3):529-43.

85. Li Q, Sun J, Guo L, Zang Y, Feng Z, Huang X, et al. Increased fractional anisotropy in white matter of the right frontal region in children with attention-deficit/hyperactivity disorder: a diffusion tensor imaging study. Neuroendocrinology Letters. 2010;31(6):747-53.

86. Shang CY, Wu YH, Gau SS, Tseng WY. Disturbed microstructural integrity of the frontostriatal fiber pathways and executive dysfunction in children with attention deficit hyperactivity disorder. Psychological medicine. 2013;43(5):1093-107.

87. Silk TJ, Vilgis V, Adamson C, Chen J, Smit L, Vance A, et al. Abnormal asymmetry in frontostriatal white matter in children with attention deficit hyperactivity disorder. [References]. Brain Imaging and Behavior. 2016;10(4):1080-9.

88. Wu Y-H, Gau SS-F, Lo Y-C, Tseng W-YI. White matter tract integrity of frontostriatal circuit in attention deficit hyperactivity disorder: Association with attention performance and symptoms. [References]. Human Brain Mapping. 2014;35(1):199-212.

89. Zhan C, Liu Y, Wu K, Gao Y, Li X. Structural and Functional Abnormalities in Children with Attention-Deficit/Hyperactivity Disorder: A Focus on Subgenual Anterior Cingulate Cortex. Brain Connect. 2017;7(2):106-14.

90. Cao Q, Shu N, An L, Wang P, Sun L, Xia M-R, et al. Probabilistic diffusion tractography and graph theory analysis reveal abnormal white matter structural connectivity networks in drug-naive boys with attention deficit/hyperactivity disorder. [References]. The Journal of Neuroscience. 2013;33(26):10676-87.

91. Beare R, Adamson C, Bellgrove MA, Vilgis V, Vance A, Seal ML, et al. Altered structural connectivity in ADHD: a network based analysis. Brain Imaging Behav. 2017;11(3):846-58.

92. Griffiths KR, Braund TA, Kohn MR, Clarke S, Williams LM, Korgaonkar MS. Structural brain network topology underpinning ADHD and response to methylphenidate treatment. Transl Psychiatry. 2021;11(1):150.

93. Gehricke JG, Kruggel F, Thampipop T, Alejo SD, Tatos E, Fallon J, et al. The brain anatomy of attention-deficit/hyperactivity disorder in young adults - a magnetic resonance imaging study. PLoS One. 2017;12(4):e0175433.

94. Li D, Li T, Niu Y, Xiang J, Cao R, Liu B, et al. Reduced hemispheric asymmetry of brain anatomical networks in attention deficit hyperactivity disorder. Brain Imaging Behav. 2019;13(3):669-84.

95. Sidlauskaite J, Caeyenberghs K, Sonuga-Barke E, Roeyers H, Wiersema JR. Whole-brain structural topology in adult attention-deficit/hyperactivity disorder: Preserved global - disturbed local network organization. NeuroImage Clinical. 2015;9:506-12.

96. Li D, Cui X, Yan T, Liu B, Zhang H, Xiang J, et al. Abnormal Rich Club Organization in Hemispheric White Matter Networks of ADHD. J Atten Disord. 2021;25(9):1215-29.

97. Mazzetti C, Gonzales Damatac C, Sprooten E, Ter Huurne N, Buitelaar JK, Jensen O. Dorsal-to-ventral imbalance in the superior longitudinal fasciculus mediates methylphenidate's effect on beta oscillations in ADHD. Psychophysiology. 2022:e14008.

98. Kölle M, Mackert S, Heckel K, Philipsen A, Ulrich M, Grön G. Lower fractional anisotropy of the corticothalamic tract and increased response time variability in adult patients with ADHD. J Psychiatry Neurosci. 2022;47(2):E99-e108.

99. Konrad A, Dielentheis TF, El Masri D, Bayerl M, Fehr C, Gesierich T, et al. Disturbed structural connectivity is related to inattention and impulsivity in adult attention deficit hyperactivity disorder. European Journal of Neuroscience. 2010;31(5):912-9.

100. Chiang H-L, Hsu Y-C, Shang C-Y, Tseng W-YI, Gau SS-F. White matter endophenotype candidates for ADHD: A diffusion imaging tractography study with sibling design. [References]. Psychological Medicine. 2020;50(7):1203-13.

101. Cooper M, Thapar A, Jones DK. ADHD severity is associated with white matter microstructure in the subgenual cingulum. NeuroImage Clinical. 2015;7:653-60.

102. Bu X, Yang C, Liang K, Lin Q, Lu L, Zhang L, et al. Quantitative tractography reveals changes in the corticospinal tract in drug-naïve children with attention-deficit/hyperactivity disorder. J Psychiatry Neurosci. 2020;45(2):134-41.

103. Chaim-Avancini TM, Doshi J, Zanetti MV, Erus G, Silva MA, Duran FLS, et al. Neurobiological support to the diagnosis of ADHD in stimulant-naïve adults: pattern recognition analyses of MRI data. Acta Psychiatr Scand. 2017;136(6):623-36.

104. Wang L, Fan GG, Yu B, Ma HW, Pan XX, Guo QY. Correlated study between reaction time and fractional anisotropy for ADHD children. [Chinese]. Chinese Journal of Medical Imaging Technology. 2008;24(4):489-92.

105. Tsai CJ, Lin HY, Tseng IW, Gau SS. White matter microstructural integrity correlates of emotion dysregulation in children with ADHD: A diffusion imaging tractography study. Prog Neuropsychopharmacol Biol Psychiatry. 2021;110:110325.

106. Langevin LM, Macmaster FP, Crawford S, Lebel C, Dewey D. Common white matter microstructure alterations in pediatric motor and attention disorders. Journal of Pediatrics. 2014;164(5):1157-64.e1.

107. Tung YH, Lin HY, Chen CL, Shang CY, Yang LY, Hsu YC, et al. Whole Brain White Matter Tract Deviation and Idiosyncrasy From Normative Development in Autism and ADHD and Unaffected Siblings Link With Dimensions of Psychopathology and Cognition. Am J Psychiatry. 2021:appiajp202020070999.

108. Hyde C, Sciberras E, Efron D, Fuelscher I, Silk T. Reduced fine motor competence in children with ADHD is associated with atypical microstructural organization within the superior longitudinal fasciculus. Brain Imaging Behav. 2021;15(2):727-37.

109. Shaw P, Sudre G, Wharton A, Weingart D, Sharp W, Sarlls J. White matter microstructure and the variable adult outcome of childhood attention deficit hyperactivity disorder. Neuropsychopharmacology. 2015;40(3):746-54.

110. Chiang HL, Yang LK, Chen YJ, Hsu YC, Lo YC, Tseng WYI, et al. Altered White-matter Tract Property in Adults with Attention-deficit Hyperactivity Disorder. Neuroscience. 2022;487:78-87.

111. Damatac CG, Chauvin RJM, Zwiers MP, van Rooij D, Akkermans SEA, Naaijen J, et al. White Matter Microstructure in Attention-Deficit/Hyperactivity Disorder: A Systematic Tractography Study in 654 Individuals. Biol Psychiatry Cogn Neurosci Neuroimaging. 2020.

112. Stephens K, Silk TJ, Anderson V, Hazell P, Enticott PG, Sciberras E. Associations Between Limbic System White Matter Structure and Socio-Emotional Functioning in Children with ADHD + ASD. J Autism Dev Disord. 2021;51(8):2663-72.

113. Chen L, Huang X, Lei D, He N, Hu X, Chen Y, et al. Microstructural abnormalities of the brain white matter in attention-deficit/hyperactivity disorder. [References]. Journal of Psychiatry & Neuroscience. 2015;40(4):280-7.

114. Graziano PA, Garic D, Dick AS. Individual differences in white matter of the uncinate fasciculus and inferior fronto-occipital fasciculus: possible early biomarkers for callous-unemotional behaviors in young children with disruptive behavior problems. J Child Psychol Psychiatry. 2022;63(1):19-33.

115. Konrad A, Dielentheis TF, El Masri D, Dellani PR, Stoeter P, Vucurevic G, et al. White matter abnormalities and their impact on attentional performance in adult attention-deficit/hyperactivity disorder. European Archives of Psychiatry & Clinical Neuroscience. 2012;262(4):351-60.

116. Jones DK. The effect of gradient sampling schemes on measures derived from diffusion tensor MRI: a Monte Carlo study. Magn Reson Med. 2004;51(4):807-15.

117. Skare S, Hedehus M, Moseley ME, Li TQ. Condition number as a measure of noise performance of diffusion tensor data acquisition schemes with MRI. J Magn Reson. 2000;147(2):340-52.

118. Papadakis NG, Xing D, Houston GC, Smith JM, Smith MI, James MF, et al. A study of rotationally invariant and symmetric indices of diffusion anisotropy. Magn Reson Imaging. 1999;17(6):881-92.

119. Jones DK, Knosche TR, Turner R. White matter integrity, fiber count, and other fallacies: the do's and don'ts of diffusion MRI. Neuroimage. 2013;73:239-54.

120. Soares JM, Marques P, Alves V, Sousa N. A hitchhiker's guide to diffusion tensor imaging. Front Neurosci. 2013;7:31.

121. Roalf DR, Quarmley M, Elliott MA, Satterthwaite TD, Vandekar SN, Ruparel K, et al. The impact of quality assurance assessment on diffusion tensor imaging outcomes in a large-scale population-based cohort. Neuroimage. 2016;125:903-19.

122. Andersson JLR, Sotiropoulos SN. An integrated approach to correction for off-resonance effects and subject movement in diffusion MR imaging. Neuroimage. 2016;125:1063-78.

123. Graham MS, Drobnjak I, Zhang H. Realistic simulation of artefacts in diffusion MRI for validating post-processing correction techniques. Neuroimage. 2016;125:1079-94.

124. Mukherjee P, Chung SW, Berman JI, Hess CP, Henry RG. Diffusion tensor MR imaging and fiber tractography: technical considerations. AJNR Am J Neuroradiol. 2008;29(5):843-52.

125. Han H, Glenn AL, Dawson KJ. Evaluating Alternative Correction Methods for Multiple Comparison in Functional Neuroimaging Research. Brain Sci. 2019;9(8).

126. Jeurissen B, Descoteaux M, Mori S, Leemans A. Diffusion MRI fiber tractography of the brain. NMR Biomed. 2019;32(4):e3785.

127. Yeh CH, Jones DK, Liang X, Descoteaux M, Connelly A. Mapping Structural Connectivity Using Diffusion MRI: Challenges and Opportunities. J Magn Reson Imaging. 2021;53(6):1666-82.

128. Acer N, Dolu N, Zararsiz G, Dogan MS, Gumus K, Ozmen S, et al. Anatomical characterization of ADHD using an atlas-based analysis: A diffusion tensor imaging study. EuroBiotech J. 2017;1(1):46-56.

129. Alger JR, O'Neill J, O'Connor MJ, Kalender G, Ly R, Ng A, et al. Neuroimaging of Supraventricular Frontal White Matter in Children with Familial Attention-Deficit Hyperactivity Disorder and Attention-Deficit Hyperactivity Disorder Due to Prenatal Alcohol Exposure. Neurotox Res. 2021.

130. Ashtari M, Kumra S, Bhaskar SL, Clarke T, Thaden E, Cervellione KL, et al. Attention-deficit/hyperactivity disorder: a preliminary diffusion tensor imaging study. Biological Psychiatry. 2005;57(5):448-55.

131. Bechtel N, Kobel M, Penner I-K, Klarhofer M, Scheffler K, Opwis K, et al. Decreased fractional anisotropy in the middle cerebellar peduncle in children with epilepsy and/or attention deficit/hyperactivity disorder: A preliminary study. [References]. Epilepsy & Behavior. 2009;15(3):294-8.

132. Cao Q, Sun L, Gong G, Lv Y, Cao X, Shuai L, et al. The macrostructural and microstructural abnormalities of corpus callosum in children with attention deficit/hyperactivity disorder: A combined morphometric and diffusion tensor MRI study. [References]. Brain Research. 2010;1310:172-80.

133. Çelik Z, Çolak Ç, Di Biase MA, Zalesky A, Zorlu N, Bora E, et al. Structural connectivity in adolescent synthetic cannabinoid users with and without ADHD. Brain Imaging Behav. 2020;14(2):505-14.

134. Cha J, Fekete T, Siciliano F, Biezonski D, Greenhill L, Pliszka SR, et al. Neural Correlates of Aggression in Medication-Naive Children with ADHD: Multivariate Analysis of Morphometry and Tractography. Neuropsychopharmacology. 2015;03.

135. Choi J, Lim, M. H., Lee, C., Park, J. K., Son, J., Shim, S. H., Yu, I. K., Khang, H. S., & Jeong, B. . Comparison of Diffusion Tensor Imaging in Attention Deficit Hyperactivity Disorder Boys with or without Comorbid Tic Disorders. Journal of Korean Neuropsychiatric Association. 2008;47(5):493–502.

136. Davenport ND, Karatekin C, White T, Lim KO. Differential fractional anisotropy abnormalities in adolescents with ADHD or schizophrenia. Psychiatry Research. 2010;181(3):193-8.

137. De Zeeuw P, Mandl RC, Hulshoff Pol HE, Van Engeland H, Durston S. Decreased frontostriatal microstructural organization in attention deficit/hyperactivity disorder. Human Brain Mapping. 2012;33(8):1941-51.

138. de Zeeuw P, Schnack HG, van Belle J, Weusten J, van Dijk S, Langen M, et al. Differential brain development with low and high IQ in attention-deficit/hyperactivity disorder. PLoS ONE [Electronic Resource]. 2012;7(4).

139. Douglas PK, Gutman B, Anderson A, Larios C, Lawrence KE, Narr K, et al. Hemispheric brain asymmetry differences in youths with attention-deficit/hyperactivity disorder. Neuroimage Clin. 2018;18:744-52.

140. Fayed N, Modrego PJ, Castillo J, Davila J. Evidence of Brain Dysfunction in Attention Deficit-Hyperactivity Disorder: a Controlled Study with Proton Magnetic Resonance Spectroscopy. Academic Radiology. 2007;14(9):1029-35.

141. Fuelscher I, Hyde C, Anderson V, Silk TJ. White matter tract signatures of fiber density and morphology in ADHD. Cortex. 2021;138:329-40.

142. Hamilton LS, Levitt JG, O'Neill J, Alger JR, Luders E, Phillips OR, et al. Reduced white matter integrity in attention-deficit hyperactivity disorder. NeuroReport. 2008;19(17):1705-8.

143. Helpern JA, Adisetiyo V, Falangola MF, Hu C, Di Martino A, Williams K, et al. Preliminary evidence of altered gray and white matter microstructural development in the frontal lobe of adolescents with attention-deficit hyperactivity disorder: a diffusional kurtosis imaging study. Journal of Magnetic Resonance Imaging. 2011;33(1):17-23.

144. Hyde C, Fuelscher I, Sciberras E, Efron D, Anderson VA, Silk T. Understanding motor difficulties in children with ADHD: A fixel-based analysis of the corticospinal tract. Prog Neuropsychopharmacol Biol Psychiatry. 2021;105:110125.

145. Kobel M, Bechtel N, Specht K, Klarhofer M, Weber P, Scheffler K, et al. Structural and functional imaging approaches in attention deficit/hyperactivity disorder: Does the temporal lobe play a key role? [References]. Psychiatry Research: Neuroimaging. 2010;183(3):230-6.

146. Lawrence KE, Levitt JG, Loo SK, Ly R, Yee V, O'Neill J, et al. White matter microstructure in subjects with attention-deficit/hyperactivity disorder and their siblings. Journal of the American Academy of Child & Adolescent Psychiatry. 2013;52(4):431-40.

147. Lee S JB, Go HJ, Yang PS, Kwon MJ, Choi J. Occipito-Temporal Connectivity in Medication-Naïve ADHD Children:Preliminary Diffusion Tensor Imaging Study. J Korean Neuropsychiatr Assoc. 2009.

148. Lee D, Quattrocki Knight E, Song H, Lee S, Pae C, Yoo S, et al. Differential structure-function network coupling in the inattentive and combined types of attention deficit hyperactivity disorder. PLoS One. 2021;16(12):e0260295.

149. Lei D, Ma J, Du X, Shen G, Jin X, Gong Q. Microstructural abnormalities in the combined and inattentive subtypes of attention deficit hyperactivity disorder: a diffusion tensor imaging study. Scientific reports. 2014;4:6875.

150. Li QQ, Sun JH, Guo LT, Zang YF, Feng ZZ, Huang XQ, et al. Increased Fractional Anisotropy in White Matter of the Right Frontal Region in Children with Attention-Deficit/Hyperactivity Disorder: A Diffusion Tensor Imaging Study. Act Nerv Super Rediviva. 2010;52(3):193-9.

151. Lin Q, Bu X, Wang M, Liang Y, Chen H, Wang W, et al. Aberrant white matter properties of the callosal tracts implicated in girls with attention-deficit/hyperactivity disorder. Brain Imaging Behav. 2020;14(3):728-35.

152. Lin Q, Bu X, Chen H, Liang Y, Wang W, Yi Y, et al. Sex differences in microstructural alterations in the corpus callosum tracts in drug-naïve children with ADHD. Brain Imaging Behav. 2022.

153. Malisza KL, Buss JL, Bolster RB, de Gervai PD, Woods-Frohlich L, Summers R, et al. Comparison of spatial working memory in children with prenatal alcohol exposure and those diagnosed with ADHD; A functional magnetic resonance imaging study. [References]. Journal of Neurodevelopmental Disorders. 2012;4(1):1-20.

154. O'Neill J, O'Connor MJ, Kalender G, Ly R, Ng A, Dillon A, et al. Combining neuroimaging and behavior to discriminate children with attention deficit-hyperactivity disorder with and without prenatal alcohol exposure. Brain Imaging Behav. 2022;16(1):69-77.

155. Pavuluri MN, Yang S, Kamineni K, Passarotti AM, Srinivasan G, Harral EM, et al. Diffusion tensor imaging study of white matter fiber tracts in pediatric bipolar disorder and attention-deficit/hyperactivity disorder. [References]. Biological Psychiatry. 2009;65(7):586-93.

156. Peterson DJ, Ryan M, Rimrodt SL, Cutting LE, Denckla MB, Kaufmann WE, et al. Increased regional fractional anisotropy in highly screened attention-deficit hyperactivity disorder (ADHD). Journal of Child Neurology. 2011;26(10):1296-302.

157. Qian L, Li Y, Wang Y, Wang Y, Cheng X, Li CY, et al. Shared and Distinct Topologically Structural Connectivity Patterns in Autism Spectrum Disorder and Attention-Deficit/Hyperactivity Disorder. Frontiers in Neuroscience. 2021;15:13.

158. Qiu M-g, Ye Z, Li Q-y, Liu G-j, Xie B, Wang J. Changes of brain structure and function in ADHD children. [References]. Brain Topography. 2011;24(3-4):243-52.

159. Ray S, Miller M, Karalunas S, Robertson C, Grayson DS, Cary RP, et al. Structural and functional connectivity of the human brain in autism spectrum disorders and attention-deficit/hyperactivity disorder: A rich club-organization study. [References]. Human Brain Mapping. 2014;35(12):6032-48.

160. Silk TJ, Vance A, Rinehart N, Bradshaw JL, Cunnington R. Structural development of the basal ganglia in attention deficit hyperactivity disorder: a diffusion tensor imaging study. Psychiatry Research. 2009;172(3):220-5.

161. Sun H, Chen Y, Huang Q, Lui S, Huang X, Shi Y, et al. Psychoradiologic Utility of MR Imaging for Diagnosis of Attention Deficit Hyperactivity Disorder: A Radiomics Analysis. Radiology. 2018;287(2):620-30.

162. Tremblay LK, Hammill C, Ameis SH, Bhaijiwala M, Mabbott DJ, Anagnostou E, et al. Tracking Inhibitory Control in Youth With ADHD: A Multi-Modal Neuroimaging Approach. Front Psychiatry. 2020;11:00831.

163. Wang P, Jiang X, Chen H, Zhang S, Li X, Cao Q, et al. Assessing Fine-Granularity Structural and Functional Connectivity in Children With Attention Deficit Hyperactivity Disorder. Front Hum Neurosci. 2020;14:594830.

164. Wu W, McAnulty G, Hamoda HM, Sarill K, Karmacharya S, Gagoski B, et al. Detecting microstructural white matter abnormalities of frontal pathways in children with ADHD using advanced diffusion models. Brain Imaging Behav. 2020;14(4):981-97.

165. Xia S, Li X, Kimball AE, Kelly MS, Lesser I, Branch C. Thalamic shape and connectivity abnormalities in children with attention-deficit/hyperactivity disorder. Psychiatry Research. 2012;204(2-3):161-7.

166. Zhou X, Lin Q, Gui Y, Wang Z, Liu M, Lu H. Multimodal MR Images-Based Diagnosis of Early Adolescent Attention-Deficit/Hyperactivity Disorder Using Multiple Kernel Learning. Front Neurosci. 2021;15:710133.

167. Chaim TM, Zhang T, Zanetti MV, Da Silva MA, Louza MR, Doshi J, et al. Multimodal magnetic resonance imaging study of Treatment-Naive Adults with Attention-Deficit/Hyperactivity Disorder. PLoS ONE. 2014;9(10).

168. Chiang HL, Chen YJ, Lin HY, Tseng WI, Gau SS. Disorder-Specific Alteration in White Matter Structural Property in Adults With Autism Spectrum Disorder Relative to Adults With ADHD and Adult Controls. Hum Brain Mapp. 2017;38(1):384-95.

169. Elliott BL, D'Ardenne K, Mukherjee P, Schweitzer JB, McClure SM. Limbic and Executive Meso- and Nigro-striatal Tracts Predict Impulsivity Differences in ADHD. Biol Psychiatry Cogn Neurosci Neuroimaging. 2021.

170. Hearne LJ, Lin H-Y, Sanz-Leon P, Tseng W-YI, Gau SS-F, Roberts JA, et al. Adhd symptoms map onto noise-driven structure-function decoupling between hub and peripheral brain regions. [References]. Molecular Psychiatry. 2019(Pagination).

171. Luo YY, Halperin JM, Li XB. Anatomical substrates of symptom remission and persistence in young adults with childhood attention deficit/hyperactivity disorder. European Neuropsychopharmacology. 2020;33:117-25.

172. Luo Y, Alvarez TL, Halperin JM, Li X. Multimodal neuroimaging-based prediction of adult outcomes in childhood-onset ADHD using ensemble learning techniques. Neuroimage Clin. 2020;26:102238.

173. Makris N, Buka SL, Biederman J, Papadimitriou GM, Hodge SM, Valera EM, et al. Attention and executive systems abnormalities in adults with childhood adhd: A DT-MRI study of connections. Cerebral Cortex. 2008;18(5):1210-20.

174. Schweren LJ, Hartman CA, Zwiers MP, Heslenfeld DJ, Franke B, Oosterlaan J, et al. Stimulant treatment history predicts frontal-striatal structural connectivity in adolescents with attention-deficit/hyperactivity disorder. Eur Neuropsychopharmacol. 2016;26(4):674-83.

175. Versace A, Jones NP, Joseph HM, Lindstrom RA, Wilson TK, Lima Santos JP, et al. White matter abnormalities associated with ADHD outcomes in adulthood. Mol Psychiatry. 2021.

176. Wang B, Wang GS, Wang X, Cao R, Xiang J, Yan T, et al. Rich-Club Analysis in Adults With ADHD Connectomes Reveals an Abnormal Structural Core Network. Journal of Attention Disorders. 2021;25(8):1068-79.
